# Multilocus Oxford Nanopore sequencing reveals genetic diversity of *Giardia duodenalis* and *Cryptosporidium* spp. in surface and wastewater in Southwestern Colombia

**DOI:** 10.1186/s13071-026-07565-0

**Published:** 2026-08-26

**Authors:** Stivenn Gutiérrez, Vanessa Urrea, Luz H. Patiño, Arsenio Hidalgo-Troya, Luis Alejandro Galeano, Marina Muñoz, Juan David Ramírez

**Affiliations:** 1 Centro de Investigaciones en Microbiología y Biotecnología-UR (CIMBIUR), School of Sciences and Engineering, Universidad del Rosario, Bogotá, Colombia; 2 Grupo de Investigación Salud Pública, Departamento de Matemáticas y Estadística, Universidad de Nariño, Pasto, Colombia; 3 Grupo de Investigación en Materiales Funcionales y Catálisis (GIMFC), Departamento de Química, Universidad de Nariño, Pasto, Colombia; 4 Instituto de Biotecnología-UN (IBUN), Universidad Nacional de Colombia, Bogotá, Colombia; 5 Centro de Tecnología en Salud (CETESA), Innovaseq SAS, Funza, Cundinamarca Colombia; 6 Center for Global Health and Interdisciplinary Research, USF Genomics Program, Department of Global, Environmental and Genomic Health Sciences, College of Public Health, University of South Florida, Tampa, FL USA

**Keywords:** *Giardia duodenalis*, *Cryptosporidium* spp., Oxford Nanopore Technologies (ONT), Multilocus genotyping, Waterborne pathogens, One health framework

## Abstract

**Background:**

*Giardia duodenalis* and *Cryptosporidium* spp. are globally significant enteric protozoa responsible for waterborne diarrheal disease with important public health and zoonotic implications. Conventional genotyping approaches based on Sanger sequencing frequently underestimate mixed infections and minority variants, limiting the resolution of environmental molecular surveillance. This study aimed to characterize the presence and genetic diversity of both pathogens in surface water and wastewater samples from the Río Pasto basin (Nariño, southwestern Colombia) using a multilocus amplicon-based Oxford Nanopore Technologies (ONT) sequencing strategy.

**Methods:**

A total of 102 water samples were collected over seven months (August 2022–March 2023) from three surface water sites representing the upper, middle, and lower Río Pasto basin, and one wastewater collection point. Novel primers targeting the *Cryptosporidium* 18S rRNA gene were designed and validated alongside previously published primer sets for three *G. duodenalis* loci (glutamate dehydrogenase, triose phosphate isomerase, beta giardin). PCR-positive amplicons were sequenced on a MinION Mk1C device for 72 h. Reads were quality-filtered (Q ≥ 10) and taxonomically assigned using Centrifuge against curated reference databases; assignments were considered valid at ≥ 1000 reads per taxon per sample.

**Results:**

For *G. duodenalis*, 49 of 102 samples (48.0%) produced at least one PCR-positive amplicon. Successful taxonomic assignment after ONT sequencing and quality filtering was achieved in 26/32 (81.3%), 35/40 (87.5%), and 27/33 (81.8%) samples for the *bg*, *gdh*, and *tpi *loci, respectively. In surface water, assemblage A predominated across loci [mean relative read abundance: 80.7% (bg), 53.8% (gdh)]; in wastewater, assemblage B was more prevalent at the gdh locus (mean 51.5%) while assemblage A remained dominant at bg (mean 66.7%). Assemblages C–E were detected at low relative abundances, predominantly via gdh. At the sub-assemblage level, AII was the most consistently dominant lineage across both matrices and loci; co-occurrence of multiple sub-assemblages within a single sample was detected in subsets of samples across all three markers. For *Cryptosporidium* spp., 67 of 102 samples (65.7%) were PCR-positive, of which 49 (73.1%) yielded valid species-level assignments. *C. parvum* was the most abundant species (mean relative read abundance: 40.2% in surface water, 32.9% in wastewater), followed by *C. andersoni* and *C. canis*. Co-detection of two or more species within a single sample was observed in 47 of 49 assigned samples (95.9%), most frequently involving the combination of *C. parvum*, *C. andersoni*, and *C. canis* (36.7%); this high co-detection rate may partly reflect the limited taxonomic resolution of single-locus 18S rRNA genotyping combined with the sensitivity of high-depth long-read sequencing, and should be interpreted with caution. Concurrent detection of at least one *Cryptosporidium* species and at least one *G. duodenalis* assemblage was observed in 27 of 69 assigned samples (39.1%).

**Conclusions:**

The integration of newly designed primers with multilocus ONT amplicon sequencing provides improved resolution for detecting co-occurring genotypes and species of waterborne protozoa in complex environmental matrices compared to conventional single-locus Sanger approaches. These findings document broad circulation of diverse *G. duodenalis* assemblages and *Cryptosporidium* species in the Río Pasto basin, contributing to the genomic surveillance of these pathogens in Latin America. The presence and genetic diversity data reported here may support water quality monitoring and inform risk management strategies under a One Health framework; however, conclusions regarding quantitative exposure risk require complementary data on parasite viability and concentration.

**Graphical Abstract:**

**Figure:**
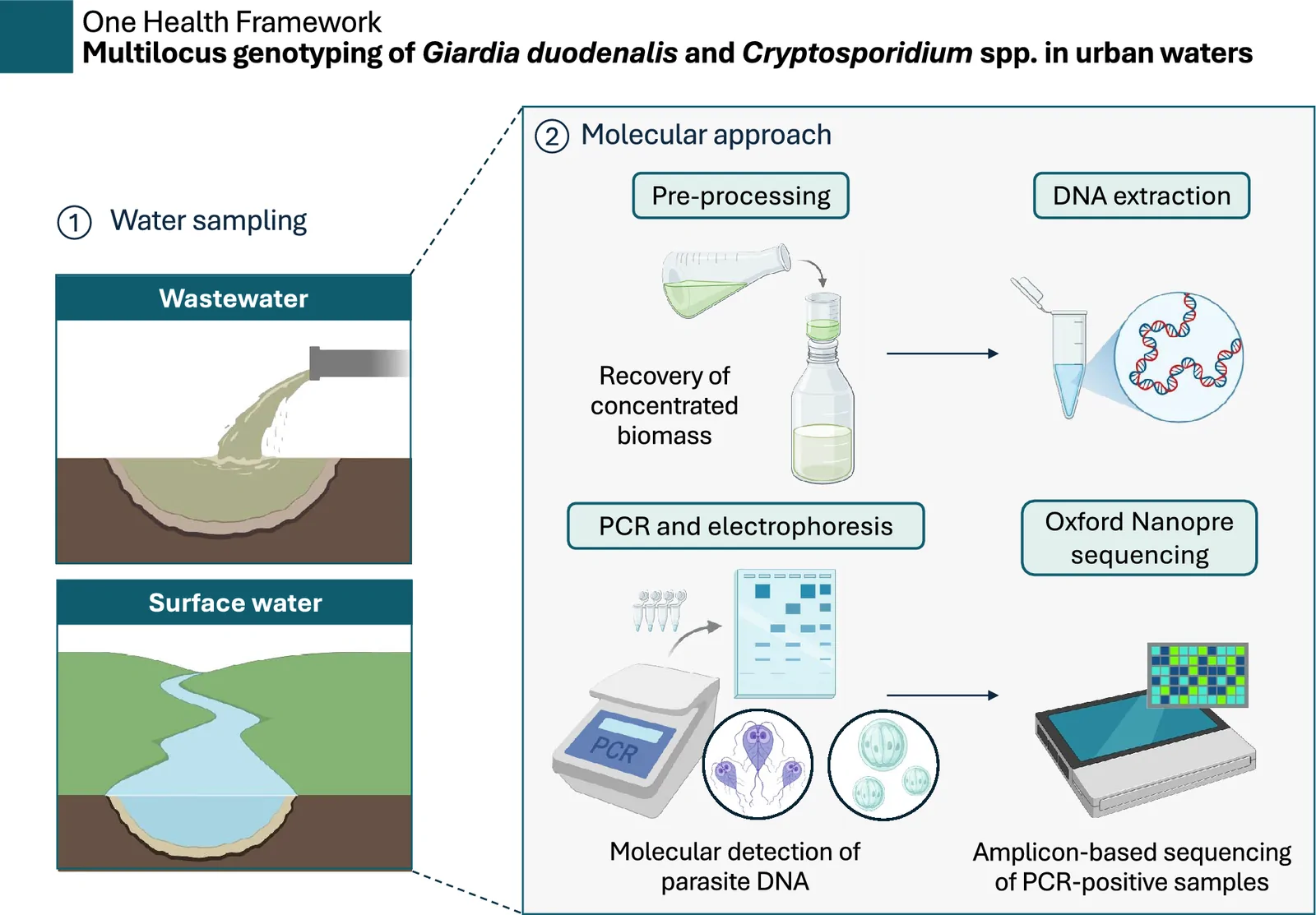

**Supplementary Information:**

The online version contains supplementary material available at https://doi.org/10.1186/s13071-026-07565-0.

## Background

*Giardia duodenalis* and *Cryptosporidium* spp. are major waterborne protozoa causing diarrheal disease globally; giardiasis affects 8–30% of children in developing regions, and cryptosporidiosis is the second leading cause of diarrheal mortality in children < 5 years [1, 2]. Transmission occurs via ingestion of cysts and oocysts in contaminated water or food; these transmissive stages can show reduced susceptibility to conventional chlorination and prolonged survival in the environment under certain conditions [1, 3]. Human-infective *G. duodenalis* assemblages A and B (sub-assemblages AI, AII, AIII, BIII, BIV) are differentiated by *gdh*, *tpi*, and *bg* loci [4]. Forty-four *Cryptosporidium* species have been described; *C. parvum* and *C. hominis* predominate in humans, and species identification relies primarily on 18S rRNA genotyping [5, 6].

*G. duodenalis* and *Cryptosporidium* spp. are globally distributed enteric protozoa with substantial genetic diversity and major roles in waterborne transmission. A recent global meta-analysis compiling 32 molecular studies from 21 countries across the Americas, Europe, Asia, and Africa reported a consistent predominance of assemblage B over A in humans across all genetic markers evaluated (*bg*, *gdh*, and *tpi*; 59.5–54.8% versus 41.6–37%) [7], and *Cryptosporidium* accounted for 77.4% of protozoan waterborne outbreaks between 2017–2022, mainly due to *C. hominis* and *C. parvum*, whereas *Giardia* represented 17.1% [8]. Environmental studies document multiple assemblages and species in surface and reclaimed waters across Brazil and Egypt [9, 10], and high environmental burdens have been confirmed in African river systems [11]. In Colombia, molecular surveys reveal broad circulation of diverse assemblages and species in human populations [12, 13]. However, routine molecular surveillance of water sources remains limited [1, 14].

Alternatively, although *G. duodenalis* and *Cryptosporidium* spp. are recognized zoonotic waterborne pathogens, their environmental genetic diversity remains incompletely resolved using third-generation sequencing [15, 16]. Conventional PCR and Sanger sequencing of SSU rRNA, *gdh*, *tpi*, *bg*, and *gp60* loci enable assemblage and subtype identification but frequently underestimate mixed infections and minority variants [17, 18]. Amplicon-based NGS has improved detection of co-infections and novel variants in animal and water matrices [15, 19, 20], yet most applications rely on short-read Illumina platforms.

Recent advances in ONT sequencing have demonstrated its utility for the genetic characterization of several protozoan parasites, particularly apicomplexans [21]. For example, multilocus ONT-based approaches have been successfully applied to genotype *Toxoplasma gondii* isolates with high resolution [22], and ONT sequencing of near full-length 18S rRNA amplicons has enabled metabarcoding of apicomplexan haemoparasites, facilitating discrimination among *Babesia*, *Hepatozoon*, *Neospora*, *Plasmodium*, *Theileria*, and *Toxoplasma* species [23, 24]. However, conventional Sanger sequencing of single loci frequently fails to detect mixed infections and minority variants in complex environmental matrices, while short-read Illumina platforms, although improving co-infection detection, provide limited haplotype-level resolution when multiple assemblages or species co-occur within a single sample [25–27]. The specific added value of long-amplicon ONT sequencing in this context lies in its capacity for direct haplotype resolution of co-occurring genotypes across multiple loci simultaneously [28], a capability that remains largely unexplored for *G. duodenalis* and *Cryptosporidium* spp. in environmental samples, particularly in Latin America, where implementation of long-read genomic surveillance for these waterborne protozoa in water systems is still lacking.

This study aimed to molecularly characterize the presence and genetic diversity of *G. duodenalis* and *Cryptosporidium* spp. in surface and wastewater samples from the Río Pasto basin (Nariño, southwestern Colombia) using third-generation sequencing on the Oxford Nanopore Technologies (ONT) platform. This approach addresses the limited genomic surveillance of these waterborne protozoa in environmental waters in Latin America and provides high-resolution data to support molecular epidemiology and public health monitoring under a One Health framework.

## Methods

### Sampling

**Fig. 1:**
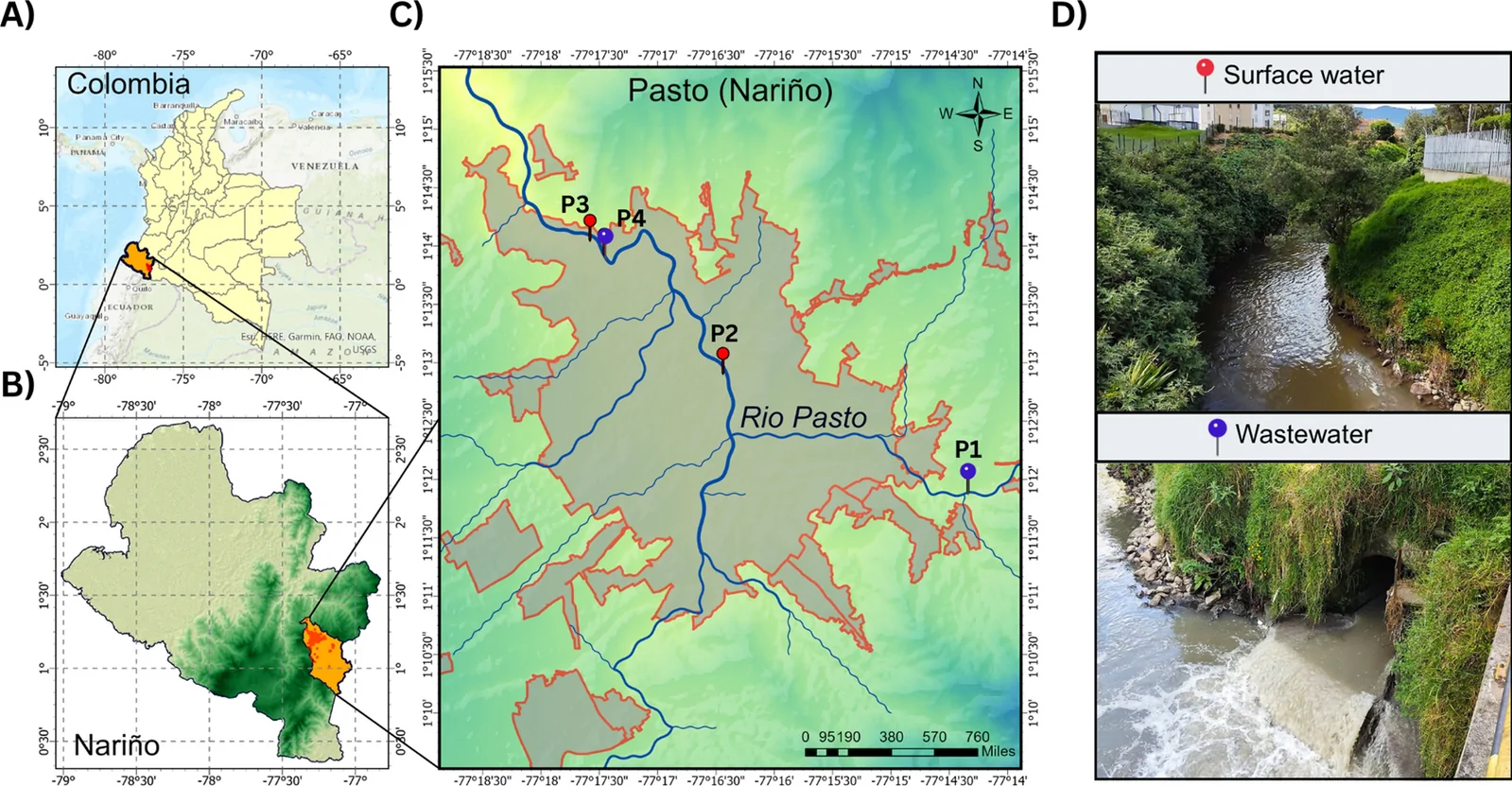
Geographic location of the sampling sites in the Pasto River basin, Nariño, Colombia. **A** Location of the Nariño department within Colombia. **B** Position of the city of Pasto within the department over a digital elevation model (DEM). **C** Course of the Pasto River in relation to the urban area. **D** Photographs of the sampling sites, showing surface-water points in red and wastewater points in blue

This study was conducted along the Río Pasto basin, the principal water resource supplying the city of San Juan de Pasto, southwestern Colombia (Fig. 1A–D). Sampling was performed over a seven-month period from August 2022 to March 2023, excluding January due to logistical constraints. San Juan de Pasto, located in the Andean highlands of the Nariño Department, represents one of the most mountainous urban areas in southern Colombia (Fig. 1A–B). Although the sample size was originally determined using EpiDat software (v.3.1) within a broader microbiological surveillance framework based on an expected bacterial diversity of 79% (5% precision, 95% confidence interval), the resulting minimum of five samples per site and water type is consistent with sample sizes reported in comparable environmental molecular surveillance studies of *G. duodenalis* and *Cryptosporidium* spp. in Latin American surface and wastewater systems [29–31]. A total of 102 water samples were collected, comprising 55 surface-water samples from three sites representing the upper (P1, *n* = 16), middle (P2, *n* = 20), and lower (P3, *n* = 19) basin, and 47 wastewater samples from the Juan XXIII collector (P4) (Fig. 1C). At each location, two collection strategies were employed using a plastic container attached to a 6 m rope. For single-sample collection, a 400 mL grab sample was taken at a specific time point. For composite sampling, 400 mL representative samples were obtained by combining equal-volume sub-samples: surface water was collected every 15 min over 1 h, while wastewater was collected hourly over 12 h, integrating temporal variability. All containers were pre-rinsed with site water prior to collection. Samples were stored in sterile glass bottles, transported to the CIMBIUR laboratory in portable coolers at 4 °C, and processed within 24 h to preserve microbial integrity [32].

### Pre-processing and DNA extraction

Biomass from the water samples was concentrated through sequential filtration using 3.0, 0.45, and 0.22 µm membrane filters (Millipore). Aliquots of 100 mL were filtered consecutively through each membrane in descending pore-size order, and each membrane was individually transferred to a separate sterile 50 mL Falcon tube. Retained material was recovered from each membrane independently by gently scraping with molecular-grade water in two 15 min rounds; eluates from all three membranes were then pooled prior to centrifugation to maximize total biomass recovery. After centrifugation, DNA was extracted from the resulting pellet using the DNeasy PowerSoil Kit (Qiagen), following the manufacturer’s instructions and employing pre-heated (65 °C) elution buffer to maximize yield. DNA concentration and purity were assessed using a Nanodrop spectrophotometer (Thermo Fisher Scientific), and DNA integrity was verified by agarose gel electrophoresis.

### Marker selection and PCR standardization for *Giardia duodenalis* and *Cryptosporidium* spp.

For *G. duodenalis*, the *gdh*, *tpi*, and *bg* loci were selected based on their widespread use and discriminatory power for assemblage and sub-assemblage characterization. Previously published primer sets targeting these loci were adopted without modification [25, 28]. Primer oligonucleotides were synthesized by Macrogen (Seoul, South Korea), and PCR conditions were optimized to ensure robust amplification and compatibility with Oxford Nanopore sequencing workflows.

For *Cryptosporidium* spp., sequences were retrieved from NCBI using the queries “*Cryptosporidium* AND 18S” and “*Cryptosporidium* AND SSUrRNA.” All sequences were aligned in UGENE, retaining only those confirmed to belong to the genus. Commonly used primers for *Cryptosporidium* detection [29] were also evaluated. Based on a manual alignment, a conserved region was identified as a candidate observation window for primer design. Primer specificity was examined using BLAST [33], and suitability was assessed with DNASP [34]. This process resulted in the design of the primers *Cryptosporidium*-F (GCGCAAATTACCCAATCCTA) and *Cryptosporidium*-R (AATACRAATGCCCCCAACTG), targeting a 486-bp region of the 18S rRNA gene. The design strategy is consistent with previously validated 18S rRNA-based primer approaches for *Cryptosporidium* species identification [35–37]. Primers were synthesized by Macrogen. PCR standardization allowed the optimization of cycling conditions (Table S1). Amplification specificity was confirmed by 2% agarose gel electrophoresis, and template DNA concentration was quantified using a Qubit fluorometer (Thermo Fisher Scientific).

### Oxford Nanopore sequencing

Amplicons testing positive by PCR were sequenced using third-generation long-read technology (Oxford Nanopore Technologies) to obtain high-quality, full-length reads. Prior to library preparation, PCR products were assessed for quality and quantity and normalized to a standard concentration of 11 ng/µL in a total volume of 11.5 µL to ensure equimolar representation across samples before barcoding. Library preparation began with DNA end-repair using the Ultra II End-Prep kit (New England Biolabs). Subsequently, barcodes were ligated to the amplicons using the Blunt/TA Ligase Master Mix (NEB), followed by attachment of Oxford Nanopore adapters through a second ligation step (Adapter Mix and Quick T4 DNA Ligase, NEB). At each stage, unincorporated reagents were removed through purification with AMPure XP paramagnetic beads (Beckman Coulter); no additional size-selection step was applied. The loading concentration of the final library was calculated based on its final concentration and the largest amplicon size among the samples to be sequenced. A total of (94) samples were pooled per run, together with a sequencing (positive) control and a negative control (reagents without genetic material), and samples were processed across two sequencing runs. The pooled library was loaded onto a FLO-MIN114 (R10.4.1) flow cell and sequenced on a MinION Mk1C device for 72 h, with run control and quality monitoring performed through the MinKNOW software suite. Raw sequencing reads generated in this study have been deposited in the European Nucleotide Archive (ENA) under project accession number PRJEB110497.

### Bioinformatic analysis

Bioinformatic processing of Nanopore reads began with basecalling using Dorado v2.0.1 by super accuracy mode, followed by quality assessment with NanoPlot v1.42.0. Reads were filtered by marker-specific length thresholds and a minimum average read quality score of Q ≥ 10 using Chopper v0.13.0.

For taxonomic assignment, custom reference databases were constructed from NCBI sequences. Searches performed in August 2025 were filtered by gene and expected size range. For *G. duodenalis*, we retrieved 735 sequences for *gdh* (500–1400 bp), 1502 for *tpi* (500–800 bp), and 1129 for *bg* (500–850 bp), representing assemblages A–H. Assemblage reference sequences were obtained from GenBank within the following accession ranges: *gdh* (AB218607.1–PV872427.1), *tpi* (AB480868.1–PV781195.1), and *bg* (AB480875.1–PV781193.1). Additionally, we incorporated sub-assemblage-specific references (AI–BIV), including *gdh* (*n* = 30; AY178738.1–MN174852.1), *tpi* (*n* = 23; EF654693.1–PP491943.1), and *bg* (*n* = 35; DQ116609.1–OQ934095.1). For *Cryptosporidium* spp., 359 sequences corresponding to the 18S rRNA gene (460–520 bp) were included. Reference sequences representing diverse species were retrieved from GenBank within the accession range EF432239.2–PX046412.1. All curated reference datasets are stored internally on the Universidad del Rosario high-performance computing cluster (Bogotá, Colombia).

Taxonomic classification of reads was performed with Centrifuge v1.0.4.2 (score ≥ 100). Taxonomic assignments were considered valid only when ≥ 1000 reads per species/sample. Co-occurrence was defined as the simultaneous taxonomic assignment of two or more species/assemblages within a single sample meeting this read-count threshold. Relative abundances were calculated for samples containing ≥ 1000 reads and visualized using ggplot2 v3.5.1 in RStudio v2025.05.0. Additional visualizations included heatmaps generated in R (ggplot2 package) and an Upset plot constructed in Python v3.11.11. Measures of dispersion were calculated from relative abundances using RStudio v2025.05.0. The complete analytical workflow is summarized in Figure S1.

## Results

### Molecular characterization of *Giardia duodenalis* by ONT sequencing

#### Locus amplification and ONT sequencing metrics

A total of 102 samples were initially screened by conventional PCR targeting the *gdh*, *tpi*, and *bg* loci. Of these, 49 (48.0%) amplified at least one locus and were selected for ONT sequencing, yielding 105 locus-specific amplicons in total. Eighteen samples were positive at all three loci. Among the remaining samples, co-amplification was observed for *bg* and *gdh* in 27 samples,* bg* and *tpi* in 21, and *gdh* and *tpi* in 26. In contrast, single-locus amplification was less frequent, detected in only a small subset of samples, including *bg* (*n* = 2), *gdh* (*n* = 5), and *tpi* (*n* = 4) (Table S2).

After quality filtering and read-cleaning procedures, ONT sequencing generated high-depth datasets for all three loci (Table S2). The *bg* marker produced 32 positive amplicons, averaging 54,135.2 reads per sample with a mean read length of 517.4 bp and a mean Q score of 17.5. The *gdh* marker yielded 40 positive amplicons, with an average of 75,990.5 reads, a mean read length of 570.3 bp, and a mean Q score of 12.8. For *tpi*, 33 positive amplicons were recovered, averaging 95,318.6 reads per sample, with a mean read length of 528.3 bp and a mean Q score of 17.5 (Table S2).

#### Assemblage and sub-assemblage composition across loci

**Fig. 2:**
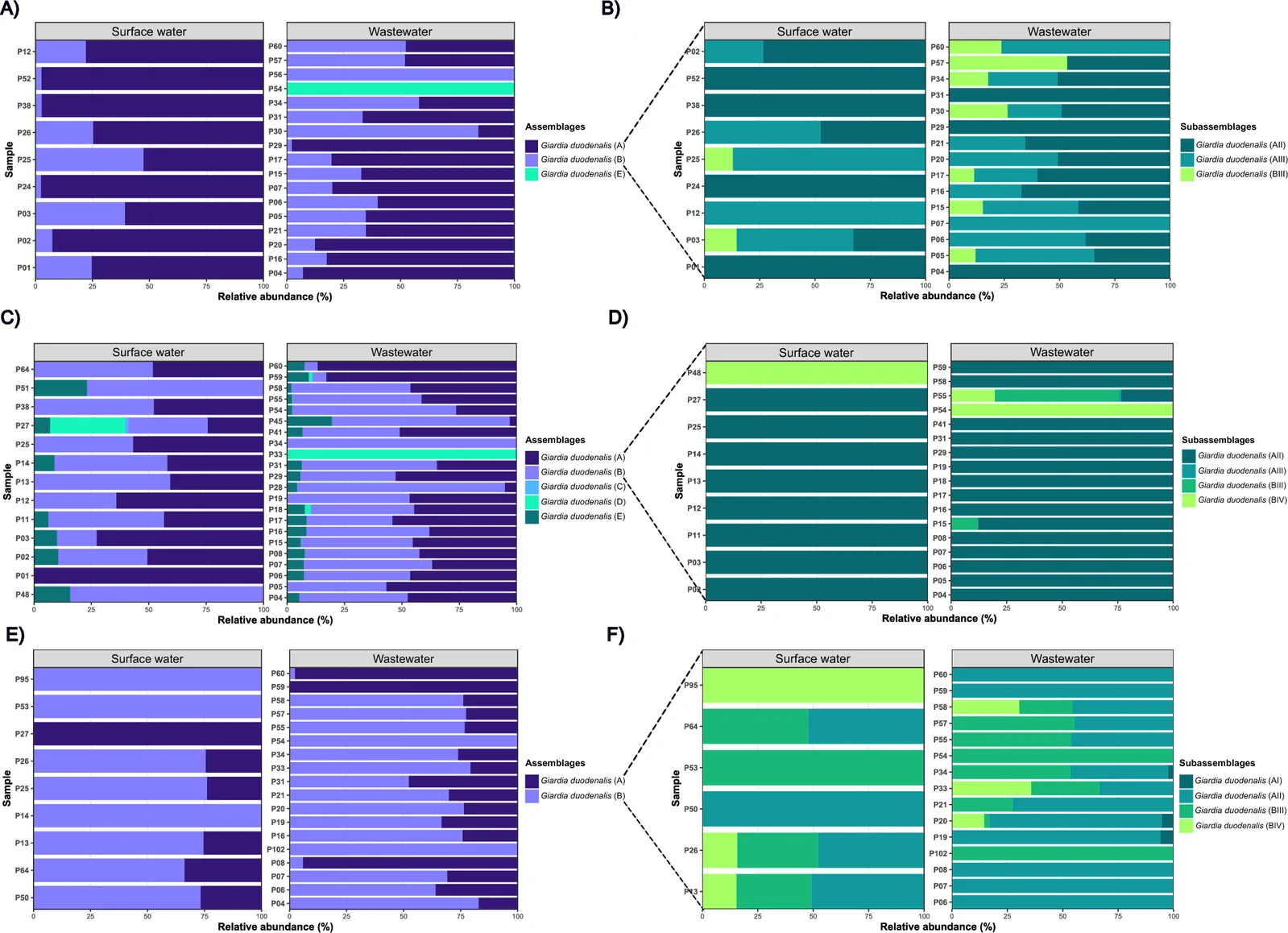
Relative abundances of *G. duodenalis* in positive samples based on the molecular markers *bg*, *gdh*, and *tpi*. Taxonomic assignment by assemblage in surface water and wastewater is shown for *gdh* (**A**–**B**), *tpi* (**C**–**D**), and *bg* (**E**–**F**)

The *bg* locus was successfully assigned in 26 of 32 samples (81.3%), revealing assemblages A, B, and E, with A and B predominating overall and a higher proportion of detections in wastewater (17/26, 65.4%) than in surface water (9/26, 34.6%) (Fig. 2A, Table S3). In surface water, assemblage A was dominant (mean 80.72% ± 16.7; median 77.9%), whereas in wastewater assemblages A and B showed comparable prevalence, with A remaining slightly predominant (mean 66.7% ± 21.8; median 66.7%) (Table S5). Assemblage E was infrequent, detected in only 1 of 26 samples (3.8%) at low prevalence (5.9%) (Table S5). At the sub-assemblage level, AII, AIII, and BIII were identified in 24 of 26 samples (92.3%), more frequently in wastewater (15/24, 62.5%) (Fig. 2B); AII was the most prevalent and stable lineage, reaching near-complete dominance in several surface water samples, while AIII persisted at moderate levels (mean ~ 56.2%) across both matrices, and BIII displayed the greatest variability in abundance (mean ~ 18.3%) (Table S5).

Of 40 *gdh*-positive samples, 35 (87.5%) were successfully genotyped by ONT sequencing (Table S3), revealing assemblages A through E, with detections predominating in wastewater (22/35, 62.9%) over surface water (13/35, 37.1%) (Fig. 2C). Assemblage A was predominant in surface water (mean 53.8% ± 19.9) and assemblage B in wastewater (mean 51.5% ± 22.1), while assemblages D and E occurred at consistently lower relative abundances across both matrices (Table S5); notably, sample P27 harbored assemblages A, B, C, D, and E simultaneously (Fig. 2C). At the sub-assemblage level, AII, AIII, BIII, and BIV were identified in 26 of 35 samples (74.3%), more frequently in wastewater (17/26, 65.4%) (Fig. 2D); AII showed the highest prevalence in both surface water and wastewater (88.9% and 94.1%, respectively), followed by BIII and BIV (Table S5). Co-occurrence of multiple sub-assemblages was observed in two samples: P15 harbored AII and BIII jointly, while P55 presented the greatest complexity, with up to four sub-assemblages detected simultaneously (Fig. 2D).

Among 33 *tpi*-positive samples, 27 (81.8%) were assigned to assemblages A and B, with detections occurring more frequently in wastewater (18/27, 66.7%) than in surface water (9/27, 33.3%) (Fig. 2E). Across both matrices, assemblage B was consistently predominant, showing higher relative abundance than A in surface water (mean 83.2% ± 14.3 vs. 39.1% ± 30.0) and in wastewater (mean 67.5% ± 26.5 vs. 40.8% ± 28.9) (Table S5). At the sub-assemblage level, AI, AII, BIII, and BIV were identified in 21 of 27 samples (77.8%), predominantly in wastewater (15/21, 71.4%); AII was the dominant lineage in both environments, reaching mean relative abundances of 62.8% ± 24.9 in surface water and 73.7% ± 27.0 in wastewater, while AI was exclusively detected in wastewater samples at low abundance (mean 4.6% ± 1.8) (Table S5). Co-occurrence of multiple sub-assemblages was limited to a subset of samples: P20 uniquely harbored all four sub-assemblages simultaneously (AI, AII, BIII, and BIV), whereas BIV was otherwise restricted to samples P13, P20, P26, P33, P58, and P95 (Fig. 2F).

#### Multilocus concordance

Among the 47 positive samples, eight (17.0%) yielded complete multilocus data for all three markers (*bg*, *gdh*, and *tpi*). Within this subset, assemblage assignments were largely concordant across loci, particularly for assemblages A and B, which showed the highest inter-locus agreement and were consistently identified in seven and eight samples, respectively (Fig. 2). Concordant assignments were defined as assemblages detected by all three loci, whereas detections supported by only one or two markers were considered incongruent across loci.

In contrast, the *gdh* marker additionally detected assemblages C, D, and E in 26 samples, indicating lower concordance with *bg* and *tpi* and highlighting marker-dependent variation in assemblage recovery. Across all markers, assemblages A and B predominated in both surface water and wastewater samples (Table S3). However, wastewater samples exhibited greater genetic complexity, characterized by more frequent detection of minority assemblages (C–E) and a higher prevalence of mixed sub-assemblage profiles within individual samples. At the sub-assemblage level, AII was consistently the dominant lineage, whereas AI, AIII, BIII, and BIV were detected less frequently but often reached high relative abundances, particularly in wastewater samples (Fig. 2).

### Molecular characterization of *Cryptosporidium* spp. by ONT sequencing

#### Locus amplification and ONT sequencing metrics

A total of 102 samples were initially screened by conventional PCR targeting the 18S rRNA locus for *Cryptosporidium* species identification, of which 67 (65.7%) yielded amplification products and were selected for ONT sequencing. Nanopore sequencing generated a mean of 26,463.9 reads per sample, with a mean read length of 531.3 bp and a mean Q score of 11.0 (Table S2).

#### Species composition and co-occurrence

**Fig. 3:**
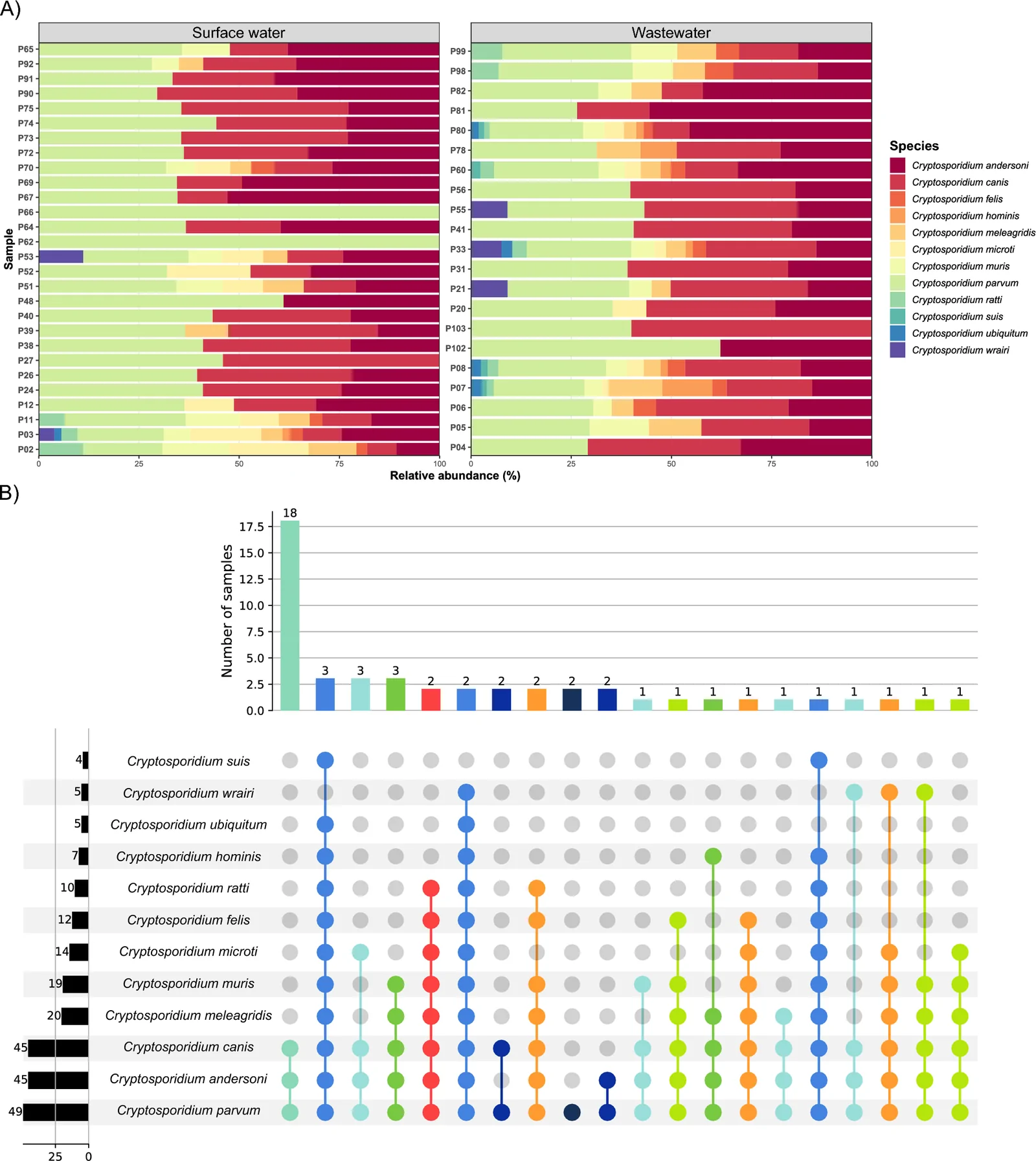
Relative abundance and co-occurrence of *Cryptosporidium* species detected using the 18S rRNA marker in surface water and wastewater. **A** Distribution by species. **B** UpSet plot showing species co-occurrences per sample

Species-level assignment was achieved in 49 of 67 samples (73.1%), with broadly similar representation across surface water (28/49, 57.1%) and wastewater (21/49, 42.9%) (Fig. 3-A, Table S4). In both matrices, *C. parvum* was the most abundant species (surface water mean 40.2% ± 18.6; wastewater mean 32.9% ± 8.6), followed by *C. andersoni* (29.1% ± 10.4; 25.2% ± 11.9) and *C. canis* (25.6% ± 12.6; 28.7% ± 12.6), whereas *C. felis*, *C. hominis*, *C. meleagridis*, *C. microti*, *C. muris*, *C. ratti*, *C. suis*, *C. ubiquitum*, and *C. wrairi* each contributed relative abundances below 10.3% (Table S5). Co-occurrence of two or more species was strikingly common, observed in 47 of 49 assigned samples (95.9%), with the combination of *C. parvum*, *C. andersoni*, and *C. canis* representing the most frequent assemblage, detected in 36.7% of samples (18/49) (Fig. 3-B).

### Co-occurrence of *Cryptosporidium* species and *G. duodenalis* genotypes

**Fig. 4:**
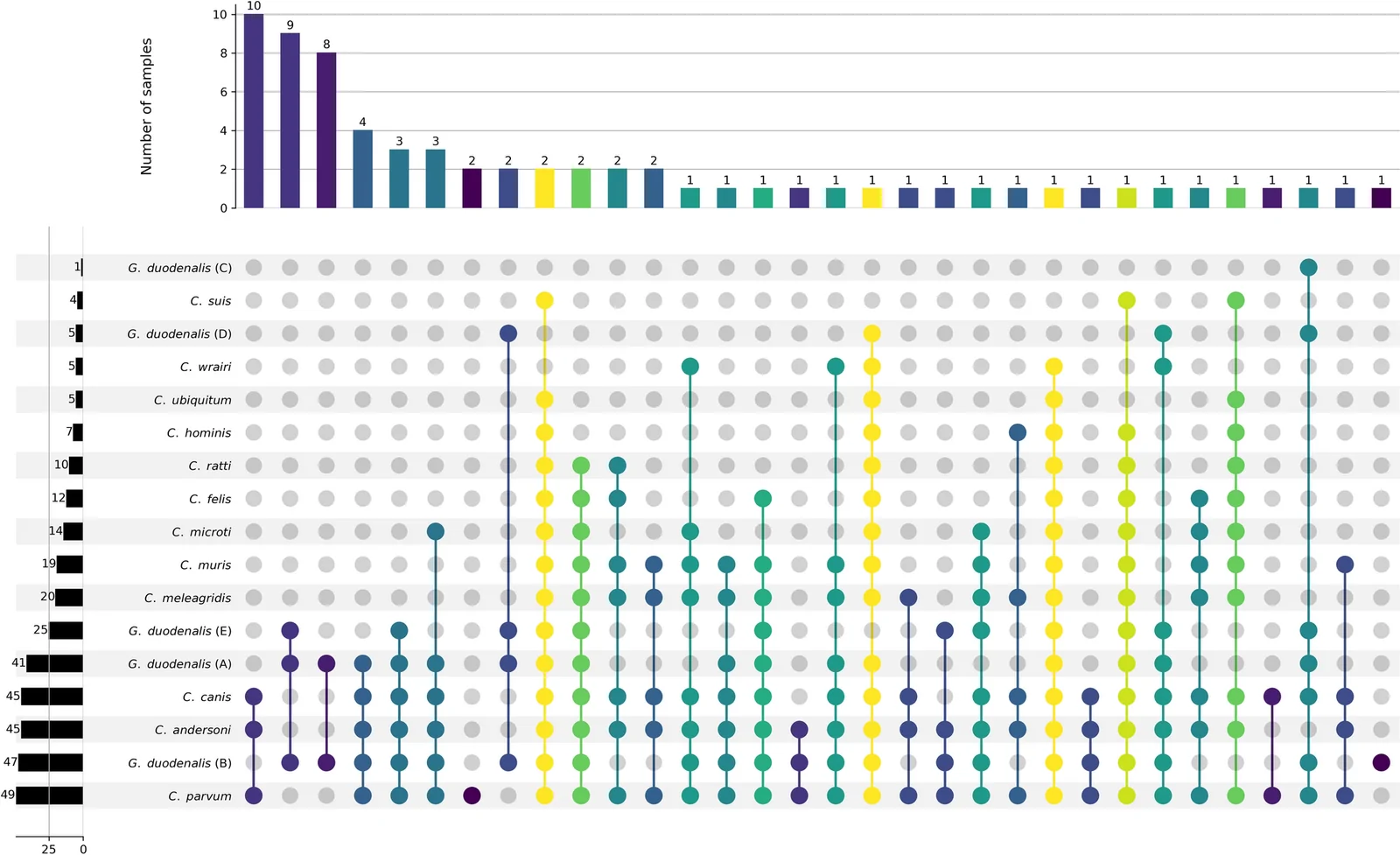
UpSet plot showing the co-occurrence of *Cryptosporidium* species and *Giardia duodenalis* assemblages across samples

Concurrent detection of at least one *Cryptosporidium* species (18S rRNA) and any *G. duodenalis* assemblage (*bg*, *gdh*, or *tpi*) was observed in 27 of 69 samples (39.1%), with genotypes considered present when supported by at least one locus (Table S6). Across matrices, *Cryptosporidium* profiles were broadly comparable, consistently dominated by *C. parvum*, *C. andersoni*, and *C. canis*, whose co-occurrence was detected in 10 of 69 samples (16.7%) distributed across both surface water and wastewater (Fig. 4). Multi-species co-occurrence was otherwise pervasive, affecting 66 of 69 assigned samples (95.7%), frequently involving *C. parvum*, *C. andersoni*, and *C. canis* alongside recurrent co-detection of *G. duodenalis* assemblages A and B; only 3 samples (4.3%) were assigned a single *Cryptosporidium* species or *G. duodenalis* assemblage exclusively (Table S6).

## Discussion

### A unified long-read framework for identifying waterborne protozoa

A multilocus strategy optimized for long-read sequencing using ONT enabled high-resolution genotypic characterization of *Giardia duodenalis*, improving discriminatory power compared with previously reported Illumina- and Sanger-based approaches [38, 39]. The integration of *gdh*, *bg*, and *tpi* loci increased detection sensitivity and enabled the resolution of mixed assemblage and sub-assemblage profiles within individual samples, addressing the well-recognized limitation of diversity underestimation inherent to single-locus genotyping [7, 40]. However, marker performance was not uniform: *gdh* provided the broadest assemblage coverage, uniquely detecting assemblages C, D, and E that were not recovered by *bg* or *tpi*, whereas only a minority of samples (17.0%) yielded complete concordant multilocus profiles, highlighting both the complementary nature and locus-dependent resolution of the multilocus approach. At the same time, such differences may reflect a combination of true biological variation and locus-specific amplification efficiency, which should be considered when interpreting assemblage richness across markers [40]. Concurrent long-read sequencing of the 18S rRNA gene further enabled high-resolution detection of *Cryptosporidium* spp., consistent with previous nanopore-based studies demonstrating improved recovery of mixed and low-abundance genotypes [41, 42]. Collectively, this integrated long-read multilocus framework enhances fine-scale resolution of enteropathogenic protozoa in aquatic environments, while also emphasizing the importance of cautious interpretation of locus-dependent detection patterns in molecular surveillance under a One Health framework.

The study was conducted in Pasto, a mid-sized Andean city located in the Department of Nariño, southwestern Colombia (1° 12′ 52″ N, 77° 16′ 41″ W; 2,527 m above sea level; mean temperature ~ 12 °C), a high-altitude peri-urban setting characterized by mixed agricultural and urban land use [29]. Surface water samples were collected at four points along the main river corridor traversing the city — from an upstream agricultural catchment (P1) through the urban stretch (P2–P3) to a major untreated wastewater discharge point (P4)—capturing a gradient of anthropogenic pressures including agricultural runoff, urban stormwater, and direct sewage inputs [43]. The absence of centralized wastewater treatment prior to discharge into the river system, combined with peri-urban livestock activity and dense informal settlements, creates conditions favorable for the sustained introduction of enteric protozoa into surface waters.

### *Giardia duodenalis* assemblage and sub-assemblage diversity

Assemblages A and B predominated in surface water and wastewater, consistent with their established zoonotic relevance and indicating sustained circulation of broad-host-range genotypes linked to fecal contamination [44, 45]. The high detection frequencies of *G. duodenalis* and *Cryptosporidium* spp. relative to prior reports [46, 47] likely reflect the enhanced analytical sensitivity of long-read sequencing using ONT. These findings demonstrate that third-generation sequencing enables sensitive, assemblage-level characterization of enteric protozoa in complex aquatic matrices, strengthening molecular surveillance capacity in settings with elevated public health vulnerability.

The *bg* marker resolved 81.3% of samples into assemblages A, B, and E, with A and B predominating and wastewater accounting for the majority of detections (65.4%). Assemblage A dominated in surface water (mean 80.7%), while assemblages A and B showed comparable prevalence in wastewater, consistent with prior multilocus surveys reporting similar distribution patterns across markers [7]. At the sub-assemblage level, AII, AIII, and BIII were identified in 92.3% of genotyped samples; AII was the most prevalent and stable lineage across both matrices, AIII persisted at moderate levels (mean ~ 56.2%), and BIII showed the greatest variability in abundance (mean ~ 18.3%). The recurrent dominance of AII across water types aligns with patterns observed in wastewater surveillance systems [48] and indicates stable circulation of clinically relevant genotypes in impacted aquatic environments. Integration of *bg* within a multilocus framework thus refines assemblage-level resolution and strengthens molecular surveillance of enteric protozoa in urban water systems.

The *gdh* marker provided the broadest assemblage resolution, recovering assemblages A through E in 87.5% of genotyped samples, with detections predominating in wastewater (62.9%) over surface water (37.1%). Assemblage A was predominant in surface water (mean 53.8%) while assemblage B prevailed in wastewater (mean 51.5%), confirming the discriminatory capacity of *gdh* for environmental genotyping; notably, sample P27 simultaneously harbored all five assemblages, illustrating the exceptional intra-sample complexity resolvable by long-read sequencing. Their relative abundance and distribution align with prior molecular surveys [38, 49] and support sustained circulation of broad-host-range genotypes in anthropized aquatic systems. At the sub-assemblage level, AII, AIII, BIII, and BIV were identified in 74.3% of genotyped samples, more frequently in wastewater (65.4%), with AII showing the highest prevalence across both surface water (88.9%) and wastewater (94.1%). Co-occurrence of multiple sub-assemblages—including up to four simultaneously in sample P55—confirms that long-read sequencing enables fine-scale resolution of clinically relevant genotypes that short-read and single-locus methods typically underestimate [50, 51]. Collectively, these findings establish *gdh* as the most informative single marker and underscore the value of multilocus frameworks for tracking assemblage diversity and mixed-subtype circulation in contaminated aquatic environments.

The *tpi* marker resolved 81.8% of samples into assemblages A and B—not 84.8% or three assemblages as previously reported—with detections more frequent in wastewater (66.7%) than surface water (33.3%), and assemblage B consistently predominating across both matrices (mean 83.2% in surface water; 67.5% in wastewater), supporting the high environmental sensitivity of this locus [52, 53]. At the sub-assemblage level, AI, AII, BIII, and BIV were identified in 77.8% of assigned samples, predominantly in wastewater (71.4%); AII was the dominant lineage in both environments (mean 62.8% in surface water; 73.7% in wastewater), while AI was exclusively detected in wastewater at low abundance (mean 4.6%), and BIV was restricted to a limited subset of samples. Sample P20 uniquely harbored all four sub-assemblages simultaneously, demonstrating the intra-sample complexity accessible through long-read approaches [54]. Although *tpi* achieved robust overall detection, integration with *gdh* and *bg *provided complementary assemblage-specific resolution—particularly for minority assemblages and rare sub-assemblages—demonstrating that a combined multilocus strategy enhances epidemiological coverage and fine-scale genotypic discrimination in complex aquatic systems [7, 55].

Detection of low-frequency assemblages (C, D, and E) expanded the known genetic diversity of *G. duodenalis*, with higher diversity observed in wastewater compared to surface water, suggesting continuous input from multiple human and animal reservoirs [56–59]. The detection of up to five assemblages and multiple sub-assemblages within individual samples, indicates substantial intra-sample complexity that is often underestimated by conventional single-marker approaches [60]. However, such patterns should be interpreted cautiously, as they may also be influenced by methodological factors including differential PCR amplification efficiency, marker-specific sensitivity, residual sequencing errors, and potential bioinformatic misclassification inherent to long-read amplicon data [61].

Despite these limitations, the consistent predominance and inter-locus concordance of assemblages A and B across markers support their robust detection and suggest active circulation of clinically relevant genotypes in impacted aquatic environments [49]. The frequent detection of additional assemblages (C–E), particularly in wastewater, likely reflects both true environmental heterogeneity and increased sensitivity of the *gdh* marker, which showed higher recovery of minority taxa compared to *bg* and *tpi* [55]. Collectively, multilocus long-read sequencing provides improved resolution of assemblage diversity and mixed-genotype patterns, although co-occurrence interpretations should be considered as putative ecological signals rather than definitive transmission linkages.

### *Cryptosporidium* species composition and environmental reservoirs

Environmental ONT-based 18S rRNA sequencing enabled species-level resolution in 73.1% of *Cryptosporidium*-positive samples (49/67), revealing pervasive multi-species co-occurrence and contributions from both human and animal reservoirs. *C. parvum* was the dominant species across both matrices, followed by *C. andersoni* and *C. canis*, and the three-species assemblage *C. parvum–C. andersoni–C. canis* represented the most frequently detected combination (36.7% of assigned samples). The prominence of *C. parvum* is consistent with its established role as the primary zoonotic and anthroponotic *Cryptosporidium* species globally, with both ruminant livestock and human sewage as principal sources [62, 63]. The co-dominance of *C. andersoni*, a bovine-adapted species, and *C. canis*, a canine-associated species, further indicates that multiple vertebrate host classes contribute to environmental contamination in the study area. Domestic and stray dog populations are frequently implicated in environmentally transmitted parasitic infections due to uncontrolled defecation, opportunistic behavior, and close human contact [64, 65], while cattle husbandry at peri-urban interfaces represents an additional pathway for *C. andersoni* and *C. parvum* discharge into surface waters [66, 67].

The concurrent detection of *C. hominis* and other less abundant non-human-adapted species, together with the predominant *G. duodenalis* subtypes AII and BIII, reflects overlapping anthropogenic and zoonotic inputs characteristic of peri-urban water systems, consistent with prior wastewater observations [9, 29, 39, 43, 44]. Similar *Cryptosporidium* profiles across surface water and wastewater confirm these interconnected systems as shared reservoirs for mixed protozoan communities. These results demonstrate that ONT-based multilocus metabarcoding provides sufficient resolution to discriminate co-circulating *Cryptosporidium* and *Giardia* species in complex environmental matrices, supporting source attribution and refined zoonotic risk assessment [14, 20, 68]. Future work integrating longitudinal sampling and metagenomic approaches would help resolve temporal dynamics, evaluate the impact of sanitation interventions, and inform targeted strategies for mitigating protozoan contamination in aquatic environments.

### Limitations and future directions

This study demonstrates that ONT sequencing provides high-resolution detection of environmental *Giardia duodenalis* and *Cryptosporidium* spp., but several methodological constraints warrant consideration when interpreting results. Taxonomic resolution and pipeline accuracy were not benchmarked against a mock community of known composition, which limits confidence in species- and genotype-level assignments, particularly for closely related taxa where ONT error profiles—including characteristic insertion and deletion errors—may introduce misclassification at the sub-assemblage level. No correction or normalization for PCR amplification bias was applied across the four target loci (*bg, gdh, tpi*, 18S rRNA), meaning that relative abundance estimates reflect amplification efficiency as well as true biological composition and should be interpreted with caution. These issues compound locus-specific sensitivity, variable read depth, and uneven amplification across samples, which may produce discrepancies in assemblage and species assignments and complicate the distinction between true co-occurrences and sequencing artifacts, especially among closely related sub-assemblages.

Beyond sequencing-specific constraints, the absence of quantitative oocyst and cyst data, heterogeneous sample sizes across individual sampling sites (P1–P4), and reliance on a predefined marker set collectively restrict assessments of pathogen load, fine-scale spatial comparisons, seasonal dynamics, and full genomic diversity. Limited spatial and temporal coverage further constrains generalizability of the community composition patterns reported here.

Future work should prioritize mock community validation to establish pipeline accuracy thresholds and quantify taxon-specific misclassification rates under realistic environmental conditions. Incorporating internal standards or spike-in controls would help decouple amplification bias from true abundance variation. Integration of quantitative assays (e.g., digital PCR or quantitative microscopy), longitudinal and spatially balanced sampling designs, and whole-genome or adaptive sequencing approaches will further enhance resolution, validate co-occurrence patterns, and strengthen environmental protozoan surveillance within a One Health framework.

## Conclusions

ONT-based multilocus sequencing revealed high-resolution genetic diversity of *G. duodenalis* (assemblages A/B, sub-assemblages AI–AIII, BIII–BIV) and *Cryptosporidium* (*C. parvum*, *C. andersoni, C. canis*), capturing co-occurrences and minority variants typically undetected by conventional methods. This approach demonstrates dual zoonotic and anthroponotic transmission, overlapping human and animal contributions, and the capacity for precise source attribution in environmental surveillance. These findings provide actionable molecular evidence to inform water quality monitoring and targeted interventions, while future studies incorporating broader spatial coverage, additional markers, longitudinal sampling, and parasitic load quantification will further refine risk assessment under a One Health framework.

## Supplementary Information

Supplementary Material 1.

Supplementary Material 2.

Supplementary Material 3.

Supplementary Material 4.

Supplementary Material 5.

Supplementary Material 6.

Supplementary Material 7.

## Data Availability

The raw sequencing data were deposited in the European Nucleotide Archive (ENA) under project accession PRJEB110497.
