# Supplementary material for: Multilocus Oxford Nanopore sequencing reveals genetic diversity of Giardia duodenalis and Cryptosporidium spp. in surface and wastewater in Southwestern Colombia
Source: Parasit Vectors. 2026 Aug 26;19:398. doi: 10.1186/s13071-026-07565-0 (PMC13587459; doi:10.1186/s13071-026-07565-0)
Supplement: Supplementary file 1 [file 13071_2026_7565_MOESM1_ESM.pdf]

Multilocus genotyping of *Giardia duodenalis* and *Cryptosporidium* spp. in urban waters

1 Sampling

Location: Rio Pasto, Nariño, Colombia (Upper, Middle, Lower basin and Juan XXIII wastewater collector).
Timeline: August 2022 - March 2023 (7 months).
Sample size: n = 102 total (55 surface water, 47 wastewater).

2 Pre-proccessing and DNA extraction

Biomass concentration: Sequential filtration (3.0 µm, 0.45 µm, 0.22 µm Millipore membranes).
Extraction: DNeasy PowerSoil Kit (Qiagen) with pre-heated (65 °C) elution buffer.
Quality control: Concentration/purity via NanoDrop and integrity via agarose gel electrophoresis.

3 Marker selection and PCR standardization

***Giardia duodenalis*:** Primer synthesis (Macrogen) & optimization.
***Cryptosporidium* spp.:** NCBI 18S-SSUrRNA sequence alignment (UGENE): manual design of novel primers and specificity checked via BLAST/DNASP (*in-silico*).
Amplification validation: 2% agarose gel electrophoresis and Qubit fluorometer quantification.

4 Oxford Nanopore long-read sequencing

Library prep: Amplicons subjected to end-repair (Ultra II End-Prep) and barcode ligation (Blunt/TA Ligase).
Adapter ligation: Sequencing adapter attachment via Quick T4 DNA Ligase.
Purification: AMPure XP paramagnetic beads.
Sequencing run: Loaded onto FLO-MIN114 (R10.4.1) flow cell; run on MinION Mk1C for 72 hours via MinKNOW.

5 Bioinformatic pipeline

Basecalling: Dorado v2.0.1 (Super Accuracy).

QA/QC: NanoPlot v1.42.0.

Filtering: Chopper v0.13.0 (length-filtered, Qscore ≥ 10).

6 Taxonomic classification

Reference databases: Custom local NCBI databases.
Alignment: Centrifuge v1.0.4.2 (score ≥ 100).
Threshold: Validity requiring ≥ 1000 reads per species/sample.

7 Statistical analysis

Metrics: Relative abundance calculation, co-occurrence profiling, dispersion measures.
Data visualization: RStudio (ggplot2) for relative abundances and heatmaps; Python v3.11.11 for Upset plots.
