# Supplementary material for: Multilocus Oxford Nanopore sequencing reveals genetic diversity of Giardia duodenalis and Cryptosporidium spp. in surface and wastewater in Southwestern Colombia
Source: Parasit Vectors. 2026 Aug 26;19:398. doi: 10.1186/s13071-026-07565-0 (PMC13587459; doi:10.1186/s13071-026-07565-0)
Supplement: Supplementary file 2 [file 13071_2026_7565_MOESM2_ESM.docx]

**Table S1.** Primers and PCR cycling conditions used for the amplification of *Giardia* spp. and *Cryptosporidium* spp. target genes.

| **Microorganism** | **Gene** | **Primer** | **Sequence (5´- 3´)** | **PCR Conditions** | **Expected Product Length (bp)** | **Reference** |  |
| --- | --- | --- | --- | --- | --- | --- | --- |
| *Giardia duodenalis* | **GDH^a^** | GDH1 | TTCCGTRTYCAGTACAACTC | - Initial denaturation: 94 °C, 5 min - Denaturation: 94 °C, 45 s (35x) - Annealing: 51 °C, 45 s (35x) - Extension: 72 °C, 45 s (35x) - Final extension: 72 °C, 10 min | 754 | (Park *et al*., 2023) |  |
|  |  |  |  |  |  |  |  |
|  |  | GDH2 | ACCTCGTTCTGRGTGGCGCA |  |  |  |  |
|  |  | GDH3 | ATGACYGAGCTYCAGAGGCACGT |  | 520 |  |  |
|  |  | GDH4 | GTGGCGCARGGCATGATGCA |  |  |  |  |
|  | **TPI^b^** | AL3543 | AAATIATGCCTGCTCGTCG | - Initial denaturation: 94 °C, 5 min - Denaturation: 94 °C, 45 s (35x) - Annealing: 51 °C, 45 s (35x) - Extension: 72 °C, 45 s (35x) - Final extension: 72 °C, 10 min | 605 | (Higuera *et al*., 2020) |  |
|  |  |  |  |  |  |  |  |
|  |  | AL3546 | CAAACCTTITCCGCAAACC |  |  |  |  |
|  |  | AL3544 | CCCTTCATCGGIGGTAACTT |  | 532 |  |  |
|  |  | AL3545 | GTGGCCACCACICCCGTGCC |  |  |  |  |
|  | **BG^a^** | G7F | AAGCCCGACGACCTCACCCGCAGTGC | Program 1: - Initial denaturation: 94 °C, 5 min - Denaturation: 94 °C, 45 s (35x) - Annealing: 65 °C, 45 s (35x) - Extension: 72 °C, 45 s (35x) - Final extension: 72 °C, 10 min Program 2: - Initial denaturation: 94 °C, 5 min - Denaturation: 94 °C, 45 s (35x) - Annealing: 55 °C, 45 s (35x) - Extension: 72 °C, 45 s (35x) - Final extension: 72 °C, 10 min | 753 | (Park *et al*., 2023) |  |
|  |  |  |  |  |  |  |  |
|  |  |  |  |  |  |  |  |
|  |  |  |  |  |  |  |  |
|  |  |  |  |  |  |  |  |
|  |  |  |  |  |  |  |  |
|  |  |  |  |  |  |  |  |
|  |  |  |  |  |  |  |  |
|  |  |  |  |  |  |  |  |
|  |  | G759R | GAGGCCGCCCTGGATCTTCGAGACGAC |  |  |  |  |
|  |  | G7nF | GAACGAACGAGATCGAGGTCCG |  | 511 |  |  |
|  |  | G759nR | CTCGACGAGCTTCGTGTT |  |  |  |  |
| *Cryptosporidium* spp. | **18S rRNA*** | CryptosporidiumF | GCGCAAATTACCCAATCCTA | - Initial denaturation: 94 °C, 5 min - Denaturation: 94 °C, 45 s (35x) - Annealing: 55 °C, 45 s (35x) - Extension: 72 °C, 45 s (35x) - Final extension: 72 °C, 10 min | 486 | *In silico* |  |
|  |  |  |  |  |  |  |  |
|  |  |  |  |  |  |  |  |
|  |  |  |  |  |  |  |  |
|  |  | CryptosporidiumR | AATACRAATGCCCCCAACTG |  |  |  |  |
|  |  |  |  |  |  |  |  |
