# Supplementary material for: Multilocus Oxford Nanopore sequencing reveals genetic diversity of Giardia duodenalis and Cryptosporidium spp. in surface and wastewater in Southwestern Colombia
Source: Parasit Vectors. 2026 Aug 26;19:398. doi: 10.1186/s13071-026-07565-0 (PMC13587459; doi:10.1186/s13071-026-07565-0)
Supplement: Supplementary file 3 [file 13071_2026_7565_MOESM3_ESM.docx]

**Table S2.** Sequencing statistics of filtered Oxford Nanopore reads for the bg, gdh, and tpi genes of *G. duodenalis* and the 18S rRNA gene of *Cryptosporidium* spp.

| **Sample** | **Reads** | **Bases** | **Mean length** | **Median length** | **N50** | **Mean Qscore** |
| --- | --- | --- | --- | --- | --- | --- |
| *G. duodenalis* (BG gene) | | | | | | |
| P01 | 44241 | 22889644 | 517.4 | 516 | 516 | 17.6 |
| P02 | 36625 | 18913516 | 516.4 | 516 | 516 | 17.9 |
| P03 | 126664 | 65390746 | 516.3 | 516 | 516 | 17.9 |
| P04 | 26625 | 13750874 | 516.5 | 516 | 516 | 17.9 |
| P05 | 85014 | 43956873 | 517.1 | 516 | 516 | 17.9 |
| P06 | 56686 | 29264807 | 516.3 | 516 | 516 | 17.8 |
| P07 | 27483 | 14195993 | 516.5 | 516 | 516 | 17.8 |
| P12 | 20119 | 10387109 | 516.3 | 516 | 516 | 17.7 |
| P14 | 437 | 225208 | 515.4 | 516 | 516 | 17.9 |
| P15 | 158244 | 82131028 | 519 | 516 | 516 | 17.7 |
| P16 | 91835 | 47412578 | 516.3 | 516 | 516 | 17.9 |
| P17 | 109248 | 56416659 | 516.4 | 516 | 516 | 17.8 |
| P19 | 491 | 239596 | 488 | 466 | 479 | 16 |
| P20 | 23486 | 12134010 | 516.6 | 516 | 516 | 17.7 |
| P21 | 33199 | 17143942 | 516.4 | 516 | 516 | 18 |
| P24 | 42289 | 21838470 | 516.4 | 516 | 516 | 17.8 |
| P25 | 55922 | 28853102 | 516 | 516 | 516 | 17.9 |
| P26 | 63241 | 32630501 | 516 | 516 | 516 | 17.7 |
| P29 | 82763 | 42724429 | 516.2 | 516 | 516 | 17.9 |
| P30 | 185221 | 95550594 | 515.9 | 516 | 516 | 18.1 |
| P31 | 26339 | 13599947 | 516.3 | 516 | 516 | 17.9 |
| P33 | 744 | 384476 | 516.8 | 516 | 516 | 17.7 |
| P34 | 74169 | 38251950 | 515.7 | 516 | 516 | 18 |
| P38 | 36696 | 18946604 | 516.3 | 516 | 516 | 17.8 |
| P45 | 152 | 77832 | 512.1 | 515 | 516 | 16.5 |
| P52 | 105846 | 54615167 | 516 | 516 | 516 | 17.8 |
| P54 | 2181 | 1108798 | 508.4 | 478 | 504 | 17 |
| P56 | 47470 | 24497602 | 516.1 | 516 | 516 | 18.5 |
| P57 | 12643 | 6522517 | 515.9 | 516 | 516 | 17.8 |
| P59 | 30503 | 17976792 | 589.3 | 634 | 640 | 10.9 |
| P60 | 71615 | 36931964 | 515.7 | 516 | 516 | 17.9 |
| *G. duodenalis* (GDH gene) | | | | | | |
| P01 | 4308 | 2469719 | 573.3 | 592 | 612 | 10.9 |
| P02 | 19142 | 11312237 | 591 | 619 | 628 | 10.9 |
| P03 | 25321 | 14774800 | 583.5 | 616 | 627 | 10.9 |
| P04 | 72478 | 43639195 | 602.1 | 641 | 643 | 10.9 |
| P05 | 16788 | 10064885 | 599.5 | 639 | 642 | 10.9 |
| P06 | 19521 | 11676332 | 598.1 | 638 | 642 | 11 |
| P07 | 27912 | 16339468 | 585.4 | 614 | 632 | 10.9 |
| P08 | 71113 | 41033105 | 577 | 629 | 640 | 11 |
| P11 | 30503 | 17976792 | 589.3 | 634 | 640 | 10.9 |
| P12 | 17513 | 10454581 | 597 | 629 | 637 | 10.9 |
| P13 | 18589 | 11312079 | 608.5 | 641 | 643 | 10.9 |
| P14 | 36186 | 20569290 | 568.4 | 594 | 628 | 10.8 |
| P15 | 51372 | 31015658 | 603.7 | 640 | 643 | 10.9 |
| P16 | 31211 | 17829431 | 571.3 | 594 | 630 | 11 |
| P17 | 23107 | 13677167 | 591.9 | 625 | 635 | 10.9 |
| P18 | 59400 | 35191207 | 592.4 | 627 | 637 | 10.9 |
| P19 | 20012 | 11696421 | 584.5 | 616 | 633 | 10.9 |
| P24 | 2371 | 1374616 | 579.8 | 612 | 623 | 10.9 |
| P25 | 17079 | 10163065 | 595.1 | 620 | 630 | 10.9 |
| P26 | 39 | 22271 | 571.1 | 577 | 589 | 10.9 |
| P27 | 222898 | 129382294 | 580.5 | 619 | 637 | 10.9 |
| P28 | 38960 | 22760503 | 584.2 | 631 | 641 | 10.9 |
| P29 | 28484 | 17362874 | 609.6 | 639 | 641 | 10.8 |
| P30 | 1092 | 632091 | 578.8 | 596 | 633 | 10.9 |
| P31 | 22161 | 12961597 | 584.9 | 628 | 639 | 10.8 |
| P33 | 13030 | 7536555 | 578.4 | 598 | 635 | 10.9 |
| P34 | 11466 | 6686237 | 583.1 | 618 | 635 | 10.9 |
| P38 | 8079 | 4899437 | 606.4 | 641 | 643 | 10.9 |
| P41 | 84606 | 44509228 | 526.1 | 530 | 530 | 17.6 |
| P45 | 44075 | 22616817 | 513.1 | 527 | 528 | 17.5 |
| P48 | 11660 | 6110292 | 524 | 530 | 530 | 17.4 |
| P51 | 25145 | 12829123 | 510.2 | 523 | 525 | 17.5 |
| P54 | 33197 | 17572555 | 529.3 | 530 | 530 | 17.2 |
| P55 | 1150095 | 607633075 | 528.3 | 530 | 530 | 17.6 |
| P58 | 429681 | 226964531 | 528.2 | 530 | 530 | 17.5 |
| P59 | 118091 | 62846298 | 532.2 | 530 | 531 | 18 |
| P60 | 33095 | 17417577 | 526.3 | 530 | 530 | 17.6 |
| P64 | 12397 | 6553881 | 528.7 | 530 | 530 | 17.5 |
| P75 | 111456 | 58586831 | 525.6 | 529 | 529 | 17.1 |
| *G. duodenalis* (TPI gene) | | | | | | |
| P04 | 8174 | 4321394 | 528.7 | 529 | 529 | 17.1 |
| P06 | 39670 | 20974090 | 528.7 | 530 | 530 | 17.2 |
| P07 | 123153 | 64152248 | 520.9 | 529 | 529 | 17.1 |
| P08 | 104754 | 54400332 | 519.3 | 529 | 529 | 17.3 |
| P12 | 497 | 258948 | 521 | 529 | 529 | 16.9 |
| P13 | 71974 | 37987859 | 527.8 | 530 | 530 | 17.2 |
| P14 | 3008 | 1594073 | 529.9 | 530 | 530 | 16.9 |
| P16 | 9405 | 4968872 | 528.3 | 530 | 530 | 17.2 |
| P17 | 1 | 531 | 531 | 531 | 531 | 27.1 |
| P18 | 2 | 1057 | 528.5 | 528.5 | 531 | 19.1 |
| P19 | 561413 | 294978295 | 525.4 | 529 | 529 | 17.1 |
| P20 | 571735 | 302431883 | 529 | 529 | 530 | 17.1 |
| P21 | 51242 | 27107339 | 529 | 530 | 530 | 17.1 |
| P25 | 17488 | 9243650 | 528.6 | 530 | 530 | 17.1 |
| P26 | 73654 | 38952653 | 528.9 | 529 | 529 | 17.1 |
| P27 | 8774 | 4644196 | 529.3 | 530 | 530 | 16.9 |
| P31 | 11979 | 6334649 | 528.8 | 530 | 530 | 17.1 |
| P33 | 210689 | 111585893 | 529.6 | 530 | 530 | 17.1 |
| P34 | 302022 | 159838022 | 529.2 | 530 | 530 | 17.1 |
| P48 | 12243 | 6579208 | 537.4 | 546 | 558 | 16.8 |
| P50 | 26088 | 13809831 | 529.4 | 530 | 530 | 17.2 |
| P53 | 15484 | 8168675 | 527.6 | 530 | 530 | 17.2 |
| P54 | 8504 | 4492029 | 528.2 | 530 | 530 | 17.2 |
| P55 | 31276 | 16543087 | 528.9 | 530 | 530 | 17.1 |
| P57 | 57303 | 30319864 | 529.1 | 530 | 530 | 17.1 |
| P58 | 101947 | 53926145 | 529 | 530 | 530 | 17.1 |
| P59 | 47652 | 25183282 | 528.5 | 529 | 529 | 17.2 |
| P60 | 107815 | 57020297 | 528.9 | 529 | 530 | 17 |
| P64 | 123074 | 65084000 | 528.8 | 529 | 529 | 17.1 |
| P95 | 115092 | 60902209 | 529.2 | 530 | 530 | 17.3 |
| P102 | 138766 | 73513558 | 529.8 | 530 | 530 | 17.1 |
| *Cryptosporidium* spp. (18S rRNA gene) | | | | | | |
| P02 | 58252 | 29895562 | 513.2 | 520 | 535 | 11 |
| P03 | 80194 | 42744253 | 533 | 553 | 557 | 11 |
| P04 | 20013 | 10218656 | 510.6 | 517 | 526 | 11 |
| P05 | 19768 | 10339721 | 523.1 | 535 | 550 | 10.9 |
| P06 | 48757 | 25806858 | 529.3 | 561 | 567 | 11.1 |
| P07 | 179439 | 96108939 | 535.6 | 557 | 561 | 10.9 |
| P08 | 175332 | 94649453 | 539.8 | 562 | 566 | 11 |
| P11 | 56503 | 30089872 | 532.5 | 550 | 553 | 11 |
| P12 | 23348 | 12467855 | 534 | 560 | 565 | 11.1 |
| P13 | 5704 | 3005259 | 526.9 | 555 | 560 | 11.1 |
| P15 | 246 | 124901 | 507.7 | 511 | 512 | 11.1 |
| P17 | 314 | 158980 | 506.3 | 509 | 512 | 11.2 |
| P18 | 75 | 36902 | 492 | 506 | 510 | 11.2 |
| P19 | 3344 | 1728644 | 516.9 | 520 | 528 | 11 |
| P20 | 30178 | 16071378 | 532.6 | 558 | 563 | 11 |
| P21 | 44020 | 23733244 | 539.1 | 563 | 567 | 10.9 |
| P24 | 10825 | 5821060 | 537.7 | 564 | 568 | 11 |
| P26 | 12631 | 6866146 | 543.6 | 567 | 569 | 11 |
| P27 | 9347 | 4985364 | 533.4 | 561 | 565 | 10.9 |
| P28 | 2715 | 1461360 | 538.3 | 556 | 561 | 11 |
| P29 | 5154 | 2731706 | 530 | 547 | 556 | 11.1 |
| P30 | 2257 | 1212916 | 537.4 | 559 | 564 | 11 |
| P31 | 12814 | 6866435 | 535.9 | 558 | 564 | 11 |
| P33 | 95242 | 51464077 | 540.4 | 564 | 568 | 10.9 |
| P38 | 20726 | 11262892 | 543.4 | 567 | 569 | 11 |
| P39 | 18219 | 9864994 | 541.5 | 566 | 569 | 10.9 |
| P40 | 12299 | 6642201 | 540.1 | 561 | 565 | 10.9 |
| P41 | 18188 | 9753066 | 536.2 | 546 | 558 | 11 |
| P48 | 8446 | 4572416 | 541.4 | 560 | 563 | 11 |
| P50 | 738 | 383569 | 519.7 | 519.5 | 529 | 11 |
| P51 | 26218 | 13434394 | 512.4 | 516 | 522 | 11 |
| P52 | 18327 | 9755168 | 532.3 | 550 | 553 | 11 |
| P53 | 32487 | 17131623 | 527.3 | 537 | 548 | 11 |
| P54 | 4598 | 2476701 | 538.6 | 567 | 570 | 11.1 |
| P55 | 28183 | 15069049 | 534.7 | 556 | 562 | 11 |
| P56 | 26171 | 14132100 | 540 | 564 | 567 | 11 |
| P57 | 2 | 856 | 428 | 428 | 473 | 11 |
| P58 | 4845 | 2573818 | 531.2 | 558 | 563 | 11 |
| P59 | 273 | 139715 | 511.8 | 511 | 514 | 11.1 |
| P60 | 76338 | 42366605 | 555 | 570 | 572 | 11.2 |
| P62 | 7296 | 3963006 | 543.2 | 564 | 566 | 11 |
| P64 | 9954 | 5514100 | 554 | 572 | 573 | 11.2 |
| P65 | 24149 | 12807515 | 530.4 | 543 | 554 | 11 |
| P66 | 8140 | 4459485 | 547.8 | 565 | 566 | 11.1 |
| P67 | 18742 | 10058558 | 536.7 | 560 | 563 | 11.1 |
| P68 | 9 | 4413 | 490.3 | 512 | 512 | 10.9 |
| P69 | 20428 | 10952201 | 536.1 | 561 | 563 | 11.1 |
| P70 | 41626 | 22055594 | 529.9 | 557 | 561 | 11.1 |
| P71 | 6018 | 3280281 | 545.1 | 565 | 567 | 11.1 |
| P72 | 17504 | 9343582 | 533.8 | 563 | 566 | 11 |
| P73 | 29201 | 15689013 | 537.3 | 557 | 562 | 11.1 |
| P74 | 16004 | 8796025 | 549.6 | 570 | 571 | 11 |
| P75 | 20668 | 11128687 | 538.5 | 559 | 564 | 11 |
| P78 | 31937 | 17392366 | 544.6 | 566 | 569 | 11.1 |
| P79 | 31 | 16066 | 518.3 | 515 | 517 | 11.3 |
| P80 | 93735 | 52524061 | 560.3 | 573 | 575 | 11.2 |
| P81 | 12319 | 6766536 | 549.3 | 567 | 569 | 11.1 |
| P82 | 21335 | 11671182 | 547 | 567 | 569 | 11.1 |
| P89 | 220 | 111191 | 505.4 | 510 | 511 | 11.2 |
| P90 | 25089 | 13741657 | 547.7 | 568 | 571 | 11.1 |
| P91 | 27322 | 14466336 | 529.5 | 551 | 558 | 11.1 |
| P92 | 42092 | 23280351 | 553.1 | 569 | 571 | 11.1 |
| P93 | 474 | 235863 | 497.6 | 509 | 512 | 11.1 |
| P98 | 37299 | 19604764 | 525.6 | 525 | 544 | 11 |
| P99 | 45473 | 25046823 | 550.8 | 567 | 569 | 11.1 |
| P102 | 9621 | 5316291 | 552.6 | 566 | 568 | 11.1 |
| P103 | 13868 | 7521008 | 542.3 | 563 | 565 | 11 |
