# Supplementary material for: Multilocus Oxford Nanopore sequencing reveals genetic diversity of Giardia duodenalis and Cryptosporidium spp. in surface and wastewater in Southwestern Colombia
Source: Parasit Vectors. 2026 Aug 26;19:398. doi: 10.1186/s13071-026-07565-0 (PMC13587459; doi:10.1186/s13071-026-07565-0)
Supplement: Supplementary file 4 [file 13071_2026_7565_MOESM4_ESM.docx]

**Table S3.** Number of reads taxonomically assigned per sample according to molecular markers (bg, gdh, and tpi) for *G. duodenalis* assemblages (A–H) and sub-assemblages (AI–BIV).

|  |  | **Assemblages** | | | | | | | |  | **Subassemblages** | | | | |
| --- | --- | --- | --- | --- | --- | --- | --- | --- | --- | --- | --- | --- | --- | --- | --- |
|  |  | ***A*** | ***B*** | ***C*** | ***D*** | ***E*** | ***F*** | ***G*** | ***H*** |  | ***AI*** | ***AII*** | ***AII*** | ***BIII*** | ***BIV*** |
| **bg** | Samples |  |  |  |  |  |  |  |  |  |  |  |  |  |  |
|  | P01 | 36079 | 11727 | 36 | 40 | 218 | 297 | 4 | 0 |  | 194 | 5203 | 483 | 729 | 0 |
|  | P02 | 44699 | 3556 | 21 | 23 | 138 | 155 | 0 | 0 |  | 88 | 3697 | 1323 | 10 | 0 |
|  | P03 | 111230 | 71627 | 55 | 48 | 845 | 492 | 12 | 0 |  | 374 | 6666 | 10726 | 2965 | 0 |
|  | P04 | 32557 | 2451 | 11 | 3 | 83 | 133 | 1 | 0 |  | 99 | 2638 | 925 | 6 | 0 |
|  | P05 | 69729 | 36840 | 44 | 19 | 342 | 330 | 9 | 0 |  | 276 | 4538 | 7054 | 1568 | 0 |
|  | P06 | 45687 | 30276 | 30 | 12 | 262 | 277 | 4 | 0 |  | 189 | 3038 | 4907 | 602 | 0 |
|  | P07 | 34321 | 8575 | 6 | 2 | 69 | 91 | 1 | 0 |  | 109 | 912 | 3800 | 8 | 0 |
|  | P12 | 25814 | 7313 | 13 | 9 | 64 | 68 | 1 | 0 |  | 58 | 270 | 3167 | 9 | 0 |
|  | P14 | 569 | 48 | 0 | 0 | 2 | 2 | 0 | 0 |  | 2 | 42 | 28 | 0 | 0 |
|  | P15 | 131802 | 64142 | 232 | 32 | 548 | 540 | 16 | 0 |  | 572 | 12660 | 13069 | 4639 | 0 |
|  | P16 | 100394 | 21285 | 48 | 10 | 520 | 354 | 3 | 0 |  | 552 | 8222 | 3983 | 608 | 0 |
|  | P17 | 112876 | 27422 | 42 | 35 | 401 | 463 | 11 | 0 |  | 448 | 9873 | 4679 | 1849 | 0 |
|  | P19 | 53 | 204 | 0 | 167 | 249 | 194 | 0 | 0 |  | 2 | 0 | 0 | 7 | 0 |
|  | P20 | 29679 | 4163 | 9 | 7 | 70 | 122 | 2 | 0 |  | 59 | 1623 | 1571 | 9 | 0 |
|  | P21 | 29521 | 15667 | 39 | 16 | 210 | 145 | 4 | 0 |  | 125 | 3061 | 1610 | 613 | 0 |
|  | P24 | 50558 | 1257 | 34 | 24 | 133 | 211 | 0 | 0 |  | 169 | 4980 | 385 | 12 | 0 |
|  | P25 | 35492 | 31938 | 13 | 8 | 231 | 136 | 6 | 0 |  | 108 | 816 | 7133 | 1050 | 0 |
|  | P26 | 63072 | 21318 | 27 | 7 | 300 | 225 | 5 | 0 |  | 227 | 4531 | 4994 | 795 | 0 |
|  | P29 | 99983 | 2185 | 31 | 7 | 210 | 351 | 6 | 0 |  | 289 | 11244 | 759 | 32 | 0 |
|  | P30 | 54164 | 283727 | 75 | 33 | 716 | 300 | 8 | 0 |  | 341 | 4467 | 2224 | 2412 | 0 |
|  | P31 | 19170 | 9582 | 24 | 4 | 137 | 119 | 2 | 0 |  | 108 | 2741 | 257 | 540 | 0 |
|  | P33 | 935 | 281 | 0 | 1 | 8 | 2 | 0 | 0 |  | 0 | 13 | 107 | 0 | 0 |
|  | P34 | 44370 | 61425 | 33 | 22 | 500 | 267 | 12 | 0 |  | 185 | 5332 | 3275 | 1843 | 0 |
|  | P38 | 45205 | 1252 | 82 | 15 | 120 | 191 | 2 | 0 |  | 166 | 4390 | 321 | 8 | 0 |
|  | P45 | 135 | 58 | 0 | 1 | 5 | 3 | 0 | 0 |  | 0 | 0 | 20 | 1 | 0 |
|  | P52 | 124918 | 3339 | 70 | 151 | 643 | 633 | 3 | 0 |  | 393 | 11857 | 836 | 42 | 0 |
|  | P54 | 74 | 523 | 0 | 435 | 1160 | 476 | 0 | 0 |  | 0 | 0 | 0 | 76 | 0 |
|  | P56 | 58 | 96030 | 13 | 81 | 358 | 98 | 0 | 0 |  | 0 | 1 | 0 | 868 | 0 |
|  | P57 | 7833 | 8458 | 16 | 7 | 60 | 64 | 2 | 0 |  | 48 | 1276 | 114 | 1464 | 0 |
|  | P60 | 45781 | 50082 | 22 | 79 | 544 | 307 | 3 | 0 |  | 135 | 775 | 8748 | 2704 | 0 |
| **gdh** |  |  |  |  |  |  |  |  |  |  |  |  |  |  |  |
|  |  |  |  |  |  |  |  |  |  |  |  |  |  |  |  |
|  | P01 | 1224 | 239 | 34 | 94 | 250 | 14 | 8 | 2 |  | 17 | 353 | 9 | 4 | 0 |
|  | P02 | 5344 | 4080 | 152 | 394 | 1110 | 40 | 32 | 8 |  | 90 | 1646 | 33 | 67 | 8 |
|  | P03 | 11479 | 2722 | 163 | 469 | 1589 | 35 | 16 | 7 |  | 174 | 3639 | 42 | 30 | 35 |
|  | P04 | 32605 | 32229 | 220 | 290 | 3666 | 19 | 14 | 5 |  | 337 | 11842 | 27 | 362 | 703 |
|  | P05 | 7682 | 5825 | 87 | 116 | 976 | 8 | 8 | 4 |  | 91 | 2664 | 31 | 91 | 103 |
|  | P06 | 7174 | 7107 | 68 | 136 | 1130 | 11 | 7 | 8 |  | 82 | 2582 | 5 | 100 | 15 |
|  | P07 | 8219 | 12415 | 109 | 259 | 1611 | 12 | 21 | 5 |  | 98 | 2783 | 12 | 33 | 63 |
|  | P08 | 20466 | 23985 | 479 | 450 | 3711 | 24 | 44 | 20 |  | 229 | 6812 | 19 | 345 | 54 |
|  | P11 | 10886 | 12585 | 114 | 355 | 1555 | 39 | 22 | 9 |  | 160 | 3786 | 51 | 49 | 450 |
|  | P12 | 6771 | 3777 | 60 | 406 | 993 | 29 | 15 | 10 |  | 76 | 2099 | 76 | 54 | 2 |
|  | P13 | 6814 | 9916 | 75 | 218 | 974 | 22 | 12 | 18 |  | 94 | 2413 | 48 | 376 | 19 |
|  | P14 | 8469 | 9864 | 301 | 287 | 1811 | 10 | 13 | 3 |  | 75 | 2792 | 11 | 135 | 27 |
|  | P15 | 20971 | 22485 | 265 | 353 | 2782 | 22 | 24 | 9 |  | 235 | 7698 | 19 | 33 | 1056 |
|  | P16 | 7227 | 10142 | 236 | 253 | 1584 | 11 | 10 | 17 |  | 55 | 2136 | 5 | 228 | 105 |
|  | P17 | 8824 | 6078 | 131 | 269 | 1367 | 36 | 34 | 9 |  | 132 | 2966 | 5 | 105 | 9 |
|  | P18 | 19431 | 19442 | 341 | 1169 | 3339 | 116 | 115 | 31 |  | 223 | 6310 | 213 | 414 | 31 |
|  | P19 | 6540 | 7448 | 108 | 267 | 990 | 10 | 11 | 7 |  | 76 | 2258 | 10 | 3 | 19 |
|  | P24 | 838 | 510 | 18 | 37 | 110 | 2 | 1 | 1 |  | 5 | 265 | 1 | 44 | 6 |
|  | P25 | 6592 | 4996 | 53 | 187 | 820 | 24 | 31 | 1 |  | 89 | 2381 | 11 | 17 | 103 |
|  | P26 | 13 | 15 | 0 | 2 | 1 | 0 | 0 | 0 |  | 0 | 5 | 0 | 0 | 1 |
|  | P27 | 29001 | 41253 | 1696 | 38852 | 8330 | 76 | 70 | 88 |  | 254 | 7334 | 22 | 483 | 86 |
|  | P28 | 1740 | 30224 | 318 | 311 | 1446 | 27 | 3 | 10 |  | 15 | 12 | 0 | 114 | 204 |
|  | P29 | 13006 | 10172 | 94 | 411 | 1416 | 41 | 46 | 2 |  | 148 | 4596 | 10 | 26 | 247 |
|  | P30 | 363 | 271 | 3 | 4 | 39 | 0 | 0 | 0 |  | 3 | 134 | 0 | 1 | 4 |
|  | P31 | 5533 | 9358 | 160 | 177 | 1008 | 10 | 11 | 5 |  | 47 | 1802 | 3 | 128 | 12 |
|  | P33 | 559 | 235 | 96 | 3983 | 437 | 4 | 4 | 3 |  | 4 | 3 | 1 | 0 | 0 |
|  | P34 | 442 | 9911 | 71 | 106 | 397 | 13 | 4 | 3 |  | 5 | 4 | 0 | 56 | 81 |
|  | P38 | 3796 | 4130 | 27 | 26 | 297 | 1 | 1 | 1 |  | 584 | 765 | 3 | 31 | 27 |
|  | P41 | 42007 | 34653 | 67 | 312 | 5546 | 27 | 5 | 35 |  | 69 | 2471 | 33 | 25 | 17 |
|  | P45 | 1165 | 29732 | 90 | 660 | 7487 | 67 | 127 | 11 |  | 38 | 3 | 1 | 17 | 3 |
|  | P48 | 890 | 10237 | 59 | 184 | 1913 | 41 | 38 | 13 |  | 34 | 88 | 6 | 2674 | 0 |
|  | P51 | 371 | 15568 | 41 | 335 | 4667 | 24 | 146 | 6 |  | 8 | 4 | 0 | 1 | 40 |
|  | P54 | 12585 | 34046 | 10 | 39 | 1048 | 10 | 3 | 7 |  | 14 | 970 | 4 | 7768 | 4 |
|  | P55 | 594294 | 802996 | 781 | 3171 | 31636 | 578 | 86 | 190 |  | 810 | 37076 | 1554 | 31328 | 89101 |
|  | P58 | 224771 | 251880 | 154 | 564 | 9032 | 70 | 11 | 70 |  | 238 | 13593 | 23 | 622 | 70 |
|  | P59 | 88237 | 6490 | 88 | 1631 | 10089 | 31 | 9 | 23 |  | 129 | 3345 | 43 | 0 | 1 |
|  | P60 | 22155 | 1412 | 50 | 758 | 1948 | 12 | 16 | 16 |  | 64 | 498 | 27 | 0 | 1 |
|  | P64 | 6554 | 7040 | 7 | 15 | 343 | 2 | 0 | 5 |  | 5 | 407 | 1 | 16 | 0 |
| **tpi** |  |  |  |  |  |  |  |  |  |  |  |  |  |  |  |
|  |  |  |  |  |  |  |  |  |  |  |  |  |  |  |  |
|  | P04 | 1468 | 7086 | 0 | 0 | 1 | 6 | 0 | 0 |  | 29 | 425 | 2 | 111 | 109 |
|  | P06 | 9170 | 16294 | 18 | 0 | 5 | 38 | 0 | 0 |  | 137 | 2254 | 14 | 666 | 996 |
|  | P07 | 27493 | 61164 | 338 | 7 | 42 | 352 | 0 | 0 |  | 458 | 6842 | 43 | 907 | 32 |
|  | P08 | 35301 | 2153 | 607 | 12 | 43 | 543 | 0 | 0 |  | 596 | 7719 | 46 | 438 | 0 |
|  | P12 | 51 | 458 | 0 | 0 | 1 | 0 | 0 | 0 |  | 2 | 17 | 0 | 19 | 12 |
|  | P13 | 14495 | 42145 | 21 | 0 | 16 | 53 | 0 | 0 |  | 252 | 3900 | 26 | 2592 | 1172 |
|  | P14 | 739 | 1483 | 1 | 0 | 3 | 6 | 0 | 0 |  | 10 | 191 | 1 | 88 | 26 |
|  | P16 | 1858 | 5795 | 6 | 0 | 1 | 10 | 0 | 0 |  | 25 | 490 | 1 | 304 | 123 |
|  | P18 | 0 | 1 | 0 | 0 | 0 | 0 | 0 | 0 |  | 0 | 0 | 0 | 0 | 0 |
|  | P19 | 130964 | 261060 | 319 | 13 | 99 | 643 | 0 | 0 |  | 2128 | 34044 | 186 | 965 | 152 |
|  | P20 | 139400 | 450097 | 174 | 9 | 143 | 417 | 0 | 0 |  | 2404 | 34059 | 222 | 1048 | 6356 |
|  | P21 | 10573 | 24463 | 6 | 0 | 11 | 45 | 0 | 0 |  | 161 | 2758 | 20 | 1039 | 25 |
|  | P25 | 3146 | 9986 | 5 | 0 | 3 | 10 | 0 | 0 |  | 67 | 947 | 10 | 592 | 313 |
|  | P26 | 14514 | 44635 | 7 | 1 | 12 | 42 | 0 | 0 |  | 270 | 3890 | 27 | 2962 | 1269 |
|  | P27 | 3796 | 117 | 0 | 0 | 1 | 11 | 0 | 0 |  | 56 | 717 | 8 | 0 | 0 |
|  | P31 | 2586 | 2821 | 7 | 2 | 1 | 8 | 0 | 0 |  | 46 | 750 | 0 | 236 | 312 |
|  | P33 | 33516 | 127604 | 26 | 2 | 44 | 79 | 0 | 0 |  | 441 | 8592 | 51 | 7872 | 9130 |
|  | P34 | 68192 | 192400 | 18 | 1 | 47 | 198 | 0 | 0 |  | 1025 | 17777 | 112 | 21473 | 393 |
|  | P48 | 49 | 903 | 511 | 15 | 33 | 684 | 0 | 0 |  | 1 | 1 | 0 | 297 | 0 |
|  | P50 | 5811 | 15899 | 3 | 0 | 4 | 10 | 0 | 0 |  | 86 | 1505 | 15 | 978 | 343 |
|  | P53 | 2 | 19940 | 9 | 0 | 3 | 5 | 0 | 0 |  | 0 | 0 | 0 | 2530 | 48 |
|  | P54 | 3 | 10937 | 1 | 1 | 1 | 6 | 0 | 0 |  | 0 | 0 | 0 | 1307 | 8 |
|  | P55 | 5980 | 19748 | 5 | 0 | 9 | 7 | 0 | 0 |  | 53 | 1749 | 12 | 2036 | 169 |
|  | P57 | 10826 | 36894 | 3 | 0 | 12 | 23 | 0 | 0 |  | 148 | 3015 | 21 | 3752 | 323 |
|  | P58 | 18070 | 57250 | 18 | 0 | 13 | 40 | 0 | 0 |  | 303 | 5047 | 26 | 2648 | 3357 |
|  | P59 | 18949 | 417 | 11 | 0 | 11 | 43 | 0 | 0 |  | 275 | 4950 | 22 | 0 | 0 |
|  | P60 | 45473 | 1047 | 5 | 0 | 17 | 122 | 0 | 0 |  | 661 | 12752 | 71 | 2 | 0 |
|  | P64 | 31841 | 62313 | 11 | 0 | 26 | 77 | 0 | 0 |  | 503 | 8628 | 59 | 7804 | 123 |
|  | P95 | 0 | 219991 | 4 | 2 | 21 | 2 | 0 | 0 |  | 0 | 0 | 0 | 387 | 2768 |
|  | P102 | 2 | 181730 | 6 | 4 | 22 | 1 | 0 | 0 |  | 0 | 0 | 0 | 21957 | 331 |
