# Supplementary material for: Multilocus Oxford Nanopore sequencing reveals genetic diversity of Giardia duodenalis and Cryptosporidium spp. in surface and wastewater in Southwestern Colombia
Source: Parasit Vectors. 2026 Aug 26;19:398. doi: 10.1186/s13071-026-07565-0 (PMC13587459; doi:10.1186/s13071-026-07565-0)
Supplement: Supplementary file 5 [file 13071_2026_7565_MOESM5_ESM.docx]

**Table S4.** Number of reads taxonomically assigned per sample for *Cryptosporidium* species.

|  | **Species** | | | | | | | | | | | | | | | | |
| --- | --- | --- | --- | --- | --- | --- | --- | --- | --- | --- | --- | --- | --- | --- | --- | --- | --- |
|  | *C. alticolis* | *C. andersoni* | *C. bovis* | *C. canis* | *C. cuniculus* | *C. felis* | *C. hominis* | *C. meleagridis* | *C. microti* | *C. muris* | *C. parvum* | *C. ratti* | *C. suis* | *C. tyzzeri* | *C. ubiquitum* | *C. wrairi* | *C. viatorum* |
| Samples |  |  |  |  |  |  |  |  |  |  |  |  |  |  |  |  |  |
| P02 | 10 | 5103 | 67 | 3483 | 14 | 1311 | 946 | 5677 | 9440 | 7922 | 9416 | 5264 | 448 | 62 | 608 | 462 | 0 |
| P03 | 39 | 14407 | 67 | 5671 | 29 | 1917 | 1073 | 3120 | 10440 | 3863 | 12682 | 2332 | 636 | 102 | 1041 | 2274 | 0 |
| P04 | 3 | 2076 | 10 | 2422 | 5 | 316 | 190 | 343 | 302 | 396 | 1847 | 218 | 95 | 19 | 159 | 100 | 0 |
| P05 | 5 | 1292 | 18 | 2215 | 9 | 373 | 186 | 1082 | 388 | 1217 | 2438 | 948 | 157 | 19 | 228 | 83 | 1 |
| P06 | 73 | 5056 | 29 | 8033 | 9 | 1379 | 906 | 1310 | 356 | 1125 | 7404 | 606 | 422 | 43 | 672 | 164 | 1 |
| P07 | 112 | 17495 | 162 | 25050 | 50 | 4257 | 14666 | 15697 | 1950 | 5121 | 26595 | 1962 | 1547 | 223 | 3105 | 436 | 2 |
| P08 | 71 | 14874 | 161 | 24203 | 58 | 3668 | 1497 | 3511 | 3461 | 4426 | 22531 | 2243 | 1387 | 188 | 2059 | 936 | 0 |
| P11 | 16 | 5817 | 105 | 4177 | 12 | 1086 | 806 | 2642 | 3297 | 4624 | 10322 | 2197 | 457 | 51 | 536 | 411 | 0 |
| P12 | 8 | 2946 | 28 | 1954 | 12 | 435 | 511 | 827 | 1175 | 909 | 3467 | 398 | 210 | 22 | 288 | 289 | 0 |
| P13 | 3 | 758 | 7 | 546 | 1 | 108 | 96 | 165 | 243 | 313 | 737 | 102 | 36 | 9 | 63 | 22 | 1 |
| P15 | 0 | 32 | 1 | 20 | 0 | 5 | 7 | 8 | 3 | 6 | 37 | 5 | 1 | 0 | 5 | 4 | 0 |
| P17 | 0 | 30 | 0 | 38 | 0 | 3 | 4 | 11 | 10 | 18 | 47 | 7 | 0 | 1 | 4 | 4 | 0 |
| P18 | 0 | 9 | 0 | 9 | 1 | 2 | 0 | 1 | 2 | 3 | 8 | 1 | 0 | 0 | 1 | 1 | 0 |
| P19 | 2 | 337 | 1 | 347 | 1 | 61 | 48 | 56 | 175 | 88 | 461 | 29 | 27 | 3 | 44 | 18 | 1 |
| P20 | 14 | 2933 | 27 | 3923 | 8 | 570 | 412 | 691 | 1025 | 873 | 4304 | 381 | 307 | 32 | 456 | 221 | 5 |
| P21 | 33 | 3689 | 24 | 7894 | 19 | 989 | 556 | 1097 | 361 | 1283 | 6997 | 670 | 502 | 52 | 834 | 2096 | 0 |
| P24 | 3 | 1234 | 17 | 1745 | 3 | 235 | 170 | 293 | 141 | 305 | 2064 | 163 | 134 | 16 | 146 | 213 | 0 |
| P26 | 6 | 1269 | 15 | 2256 | 3 | 295 | 290 | 419 | 207 | 380 | 2300 | 198 | 142 | 22 | 217 | 42 | 1 |
| P27 | 1 | 786 | 4 | 1422 | 3 | 128 | 87 | 196 | 220 | 260 | 1209 | 105 | 82 | 12 | 126 | 50 | 0 |
| P28 | 0 | 400 | 4 | 277 | 0 | 60 | 45 | 110 | 120 | 141 | 478 | 78 | 19 | 5 | 32 | 14 | 0 |
| P29 | 0 | 833 | 6 | 466 | 3 | 109 | 58 | 112 | 289 | 133 | 830 | 67 | 53 | 3 | 57 | 40 | 0 |
| P30 | 0 | 247 | 0 | 168 | 1 | 51 | 33 | 66 | 105 | 72 | 308 | 38 | 19 | 1 | 22 | 54 | 0 |
| P31 | 3 | 1176 | 9 | 2252 | 5 | 283 | 161 | 407 | 375 | 519 | 2192 | 301 | 124 | 19 | 199 | 171 | 0 |
| P33 | 28 | 8116 | 60 | 16077 | 33 | 1919 | 1073 | 2821 | 1743 | 3338 | 15254 | 2108 | 897 | 100 | 1575 | 4425 | 2 |
| P38 | 9 | 2207 | 32 | 3665 | 1 | 501 | 607 | 913 | 323 | 688 | 4075 | 419 | 218 | 34 | 404 | 150 | 0 |
| P39 | 10 | 1511 | 36 | 3649 | 2 | 449 | 838 | 1053 | 266 | 570 | 3567 | 318 | 194 | 31 | 366 | 479 | 2 |
| P40 | 2 | 1231 | 15 | 1915 | 3 | 280 | 527 | 655 | 168 | 333 | 2410 | 163 | 120 | 10 | 222 | 444 | 0 |
| P41 | 0 | 1579 | 51 | 3123 | 4 | 408 | 195 | 527 | 363 | 598 | 3210 | 387 | 182 | 16 | 299 | 682 | 2 |
| P48 | 4 | 1296 | 13 | 620 | 2 | 327 | 127 | 447 | 410 | 561 | 2033 | 343 | 84 | 6 | 86 | 32 | 0 |
| P50 | 0 | 122 | 1 | 56 | 0 | 16 | 10 | 38 | 33 | 47 | 128 | 28 | 4 | 0 | 6 | 19 | 0 |
| P51 | 9 | 2582 | 19 | 1604 | 11 | 706 | 482 | 1255 | 1250 | 1414 | 4230 | 920 | 139 | 38 | 280 | 910 | 0 |
| P52 | 17 | 2743 | 19 | 1286 | 7 | 399 | 414 | 546 | 1780 | 745 | 2728 | 246 | 139 | 17 | 228 | 562 | 0 |
| P53 | 28 | 4597 | 23 | 2643 | 12 | 727 | 451 | 1162 | 1955 | 1582 | 5008 | 830 | 222 | 39 | 392 | 2115 | 0 |
| P54 | 0 | 747 | 0 | 860 | 0 | 115 | 81 | 174 | 62 | 177 | 655 | 113 | 37 | 5 | 45 | 16 | 0 |
| P55 | 8 | 2604 | 24 | 5352 | 6 | 616 | 324 | 631 | 780 | 753 | 4788 | 374 | 299 | 35 | 455 | 1272 | 0 |
| P56 | 27 | 2144 | 21 | 4645 | 11 | 833 | 629 | 959 | 253 | 639 | 4469 | 369 | 232 | 33 | 387 | 284 | 0 |
| P57 | 0 | 0 | 0 | 0 | 0 | 0 | 1 | 0 | 0 | 0 | 1 | 0 | 0 | 0 | 0 | 0 | 0 |
| P58 | 10 | 389 | 3 | 828 | 1 | 137 | 120 | 153 | 71 | 125 | 778 | 64 | 53 | 3 | 73 | 56 | 0 |
| P59 | 0 | 25 | 1 | 31 | 0 | 5 | 6 | 8 | 3 | 8 | 40 | 5 | 0 | 0 | 7 | 4 | 0 |
| P60 | 128 | 16409 | 97 | 6473 | 25 | 1755 | 1279 | 2430 | 1949 | 3204 | 12818 | 1680 | 1124 | 89 | 851 | 218 | 0 |
| P62 | 0 | 836 | 13 | 618 | 2 | 174 | 107 | 441 | 197 | 489 | 1267 | 344 | 99 | 10 | 123 | 59 | 0 |
| P64 | 8 | 2232 | 10 | 1332 | 6 | 228 | 137 | 404 | 282 | 518 | 2063 | 278 | 123 | 9 | 149 | 24 | 0 |
| P65 | 5 | 3238 | 17 | 1233 | 11 | 638 | 347 | 529 | 369 | 1022 | 3050 | 353 | 210 | 37 | 303 | 132 | 4 |
| P66 | 2 | 959 | 3 | 456 | 4 | 181 | 97 | 174 | 57 | 288 | 1202 | 73 | 134 | 8 | 84 | 34 | 0 |
| P67 | 1 | 4290 | 20 | 1014 | 11 | 386 | 272 | 401 | 178 | 714 | 2807 | 200 | 215 | 12 | 209 | 123 | 0 |
| P68 | 0 | 2 | 0 | 0 | 0 | 1 | 0 | 0 | 0 | 0 | 1 | 0 | 0 | 0 | 0 | 0 | 0 |
| P69 | 2 | 4335 | 18 | 1426 | 13 | 537 | 287 | 439 | 704 | 699 | 3028 | 195 | 259 | 17 | 219 | 37 | 0 |
| P70 | 7 | 5279 | 50 | 2845 | 16 | 1138 | 543 | 1034 | 1739 | 1402 | 6264 | 554 | 430 | 39 | 432 | 489 | 0 |
| P71 | 6 | 741 | 2 | 553 | 2 | 169 | 101 | 182 | 67 | 198 | 913 | 94 | 52 | 9 | 81 | 46 | 0 |
| P72 | 17 | 2361 | 18 | 2223 | 6 | 376 | 182 | 362 | 48 | 535 | 2597 | 241 | 146 | 19 | 196 | 53 | 0 |
| P73 | 11 | 2979 | 19 | 5400 | 13 | 807 | 480 | 892 | 124 | 964 | 4622 | 480 | 255 | 25 | 343 | 48 | 0 |
| P74 | 1 | 1763 | 15 | 2467 | 7 | 470 | 234 | 638 | 314 | 721 | 3363 | 457 | 194 | 28 | 266 | 81 | 0 |
| P75 | 6 | 2017 | 21 | 3686 | 8 | 519 | 249 | 405 | 105 | 587 | 3148 | 258 | 137 | 14 | 227 | 99 | 0 |
| P78 | 1 | 3505 | 25 | 3978 | 11 | 643 | 1390 | 1680 | 284 | 960 | 4811 | 301 | 294 | 41 | 504 | 169 | 1 |
| P79 | 0 | 1 | 0 | 1 | 0 | 1 | 0 | 0 | 0 | 0 | 4 | 0 | 1 | 0 | 0 | 0 | 0 |
| P80 | 5 | 35980 | 166 | 7210 | 28 | 1847 | 1457 | 2411 | 3823 | 4209 | 18425 | 1069 | 1107 | 95 | 1479 | 52 | 1 |
| P81 | 0 | 4238 | 6 | 1374 | 5 | 336 | 132 | 288 | 325 | 502 | 2017 | 173 | 134 | 13 | 185 | 101 | 0 |
| P82 | 9 | 5777 | 18 | 1398 | 11 | 446 | 286 | 1034 | 465 | 1129 | 4342 | 780 | 209 | 28 | 275 | 56 | 0 |
| P89 | 0 | 22 | 0 | 20 | 0 | 3 | 4 | 6 | 10 | 7 | 32 | 6 | 3 | 0 | 1 | 5 | 0 |
| P90 | 3 | 5003 | 42 | 4923 | 10 | 561 | 293 | 619 | 566 | 756 | 4160 | 391 | 407 | 34 | 419 | 12 | 0 |
| P91 | 19 | 4747 | 27 | 2918 | 14 | 502 | 313 | 580 | 941 | 754 | 3834 | 285 | 246 | 29 | 258 | 125 | 0 |
| P92 | 24 | 9498 | 35 | 6137 | 19 | 972 | 904 | 1609 | 901 | 1793 | 7473 | 855 | 450 | 58 | 738 | 631 | 0 |
| P93 | 0 | 48 | 0 | 53 | 2 | 7 | 5 | 19 | 18 | 22 | 67 | 14 | 2 | 1 | 7 | 5 | 0 |
| P98 | 8 | 3530 | 19 | 5522 | 14 | 1852 | 423 | 2103 | 695 | 2614 | 8770 | 1793 | 298 | 42 | 493 | 440 | 0 |
| P99 | 26 | 4628 | 55 | 3726 | 21 | 1442 | 582 | 2442 | 810 | 2886 | 8108 | 1962 | 535 | 65 | 609 | 497 | 0 |
| P102 | 9 | 1253 | 16 | 984 | 4 | 424 | 193 | 286 | 180 | 363 | 2058 | 131 | 105 | 13 | 133 | 115 | 0 |
| P103 | 11 | 836 | 10 | 3227 | 5 | 337 | 166 | 473 | 401 | 577 | 2149 | 352 | 157 | 19 | 181 | 68 | 0 |
