# Supplementary material for: Multilocus Oxford Nanopore sequencing reveals genetic diversity of Giardia duodenalis and Cryptosporidium spp. in surface and wastewater in Southwestern Colombia
Source: Parasit Vectors. 2026 Aug 26;19:398. doi: 10.1186/s13071-026-07565-0 (PMC13587459; doi:10.1186/s13071-026-07565-0)
Supplement: Supplementary file 6 [file 13071_2026_7565_MOESM6_ESM.docx]

**Table S5.** Relative abundance statistics per sample according to molecular markers (bg, gdh, tpi and 18S rRNA) for *G. duodenalis* assemblages and sub-assemblages (A–H; AI–BIV) and *Cryptosporidium* species.

|  |  |  | **Surface water** | | | | | | | |  | **Wastewater** | | | | | | | |
| --- | --- | --- | --- | --- | --- | --- | --- | --- | --- | --- | --- | --- | --- | --- | --- | --- | --- | --- | --- |
| **Species** | **Gene** | **Assemblage / Subassemblage** | **Positive samples** | **Prevalence (%)** | **Mean** | **SD (Standard Deviation)** | **Median** | **Q1 (25th percentile)** | **Q3 (75th percentile)** | **IQR** |  | **Positive samples** | **Prevalence (%)** | **Mean** | **SD (Standard Deviation)** | **Median** | **Q1 (25th percentile)** | **Q3 (75th percentile)** | **IQR** |
| *Giardia duodenalis* | bg | Assemblage A | 9 | 100 | 80.72 | 16.71 | 77.92 | 74.74 | 97.31 | 22.57 |  | 15 | 88.24 | 66.68 | 21.8 | 66.67 | 54.11 | 81.48 | 27.37 |
| *Giardia duodenalis* | bg | Assemblage B | 9 | 100 | 19.28 | 16.71 | 22.08 | 2.69 | 25.26 | 22.57 |  | 16 | 94.12 | 37.49 | 26.86 | 33.95 | 19.03 | 52 | 32.97 |
| *Giardia duodenalis* | bg | Assemblage E | - | - | - | - | - | - | - | - |  | 1 | 5.88 | 100 | - | 100 | 100 | 100 | 0 |
| *Giardia duodenalis* | bg | Subassemblage AII | 7 | 77.78 | 79.14 | 28.64 | 100 | 60.61 | 100 | 39.39 |  | 13 | 86.67 | 61.92 | 23.74 | 51.02 | 46.57 | 67.37 | 20.8 |
| *Giardia duodenalis* | bg | Subassemblage AIII | 5 | 55.56 | 63.73 | 29.63 | 52.69 | 52.43 | 87.17 | 34.74 |  | 11 | 73.33 | 48.67 | 23.2 | 43.04 | 31.99 | 57.68 | 25.7 |
| *Giardia duodenalis* | bg | Subassemblage BIII | 2 | 22.22 | 13.7 | 1.23 | 13.7 | 13.26 | 14.13 | 0.87 |  | 7 | 46.67 | 22.81 | 14.65 | 17.64 | 13.6 | 25.05 | 11.46 |
| *Giardia duodenalis* | gdh | Assemblage A | 11 | 84.62 | 53.75 | 19.92 | 48.21 | 42.77 | 60.54 | 17.77 |  | 20 | 90.91 | 44.53 | 19.8 | 45.82 | 37.84 | 51.55 | 13.71 |
| *Giardia duodenalis* | gdh | Assemblage B | 12 | 92.31 | 49.43 | 18.28 | 49.63 | 38 | 53.9 | 15.9 |  | 21 | 95.45 | 51.46 | 22.11 | 49.8 | 43.13 | 56.07 | 12.95 |
| *Giardia duodenalis* | gdh | Assemblage D | - | - | - | - | - | - | - | - |  | 4 | 18.18 | 26.11 | 49.27 | 2.11 | 1.2 | 27.02 | 25.82 |
| *Giardia duodenalis* | gdh | Assemblage E | 7 | 53.85 | 11.66 | 5.9 | 10.06 | 7.99 | 13.14 | 5.15 |  | 18 | 81.82 | 6.9 | 3.86 | 6.99 | 5.45 | 7.7 | 2.25 |
| *Giardia duodenalis* | gdh | Subassemblage AII | 8 | 88.89 | 100 | 0 | 100 | 100 | 100 | 0 |  | 16 | 94.12 | 94.45 | 19.21 | 100 | 100 | 100 | 0 |
| *Giardia duodenalis* | gdh | Subassemblage AIII | - | - | - | - | - | - | - | - |  | 1 | 5.88 | 0.98 |  | 0.98 | 0.98 | 0.98 | 0 |
| *Giardia duodenalis* | gdh | Subassemblage BIII | - | - | - | - | - | - | - | - |  | 2 | 11.76 | 34.04 | 31.08 | 34.04 | 23.05 | 45.03 | 21.98 |
| *Giardia duodenalis* | gdh | Subassemblage BIV | 1 | 11.11 | 100 |  | 100 | 100 | 100 | 0 |  | 2 | 11.76 | 59.85 | 56.78 | 59.85 | 39.77 | 79.92 | 40.15 |
| *Giardia duodenalis* | tpi | Assemblage A | 6 | 66.67 | 39.11 | 30.04 | 26.18 | 24.8 | 32.06 | 7.25 |  | 16 | 88.89 | 40.78 | 28.98 | 28.17 | 23.55 | 38.97 | 15.42 |
| *Giardia duodenalis* | tpi | Assemblage A | 8 | 88.89 | 83.17 | 14.26 | 75.75 | 74.11 | 100 | 25.89 |  | 17 | 94.44 | 67.51 | 26.52 | 75.72 | 66.59 | 77.31 | 10.72 |
| *Giardia duodenalis* | tpi | Subassemblage AI | - | - | - | - | - | - | - | - |  | 3 | 20 | 4.64 | 1.82 | 5.48 | 4.01 | 5.68 | 1.67 |
| *Giardia duodenalis* | tpi | Subassemblage AII | 4 | 66.67 | 62.82 | 24.86 | 51.7 | 50.14 | 64.38 | 14.24 |  | 13 | 86.67 | 73.73 | 27.03 | 77.64 | 45.67 | 100 | 54.33 |
| *Giardia duodenalis* | tpi | Subassemblage AIII | - | - | - | - | - | - | - | - |  | 9 | 60 | 49.67 | 33.32 | 53.32 | 27.36 | 55.45 | 28.08 |
| *Giardia duodenalis* | tpi | Subassemblage BIII | 4 | 66.67 | 54.45 | 30.94 | 41.98 | 35.81 | 60.62 | 24.81 |  | - | - | - | - | - | - | - | - |
| *Giardia duodenalis* | tpi | Subassemblage BIV | 3 | 50 | 43.64 | 48.81 | 15.63 | 15.46 | 57.81 | 42.35 |  | 3 | 20 | 26.85 | 11.02 | 30.37 | 22.43 | 33.02 | 10.59 |
| *Cryptosporidium andersoni* | 18S | - | 25 | 89.29 | 29.05 | 10.4 | 24.49 | 22.19 | 35.83 | 13.64 |  | 20 | 95.24 | 25.18 | 11.95 | 20.38 | 17.3 | 32.89 | 15.59 |
| *Cryptosporidium canis* | 18S | - | 25 | 89.29 | 25.56 | 12.58 | 23.67 | 14.43 | 34.95 | 20.52 |  | 20 | 95.24 | 28.68 | 12.62 | 28.18 | 20.32 | 38.18 | 17.86 |
| *Cryptosporidium felis* | 18S | - | 4 | 14.29 | 3.74 | 1.37 | 3.22 | 3.07 | 3.89 | 0.82 |  | 8 | 38.1 | 4.46 | 1.57 | 4 | 3.5 | 5.69 | 2.19 |
| *Cryptosporidium hominis* | 18S | - | - | - | - | - | - | - | - | - |  | 6 | 28.57 | 4.93 | 4.66 | 2.22 | 1.84 | 7.44 | 5.6 |
| *Cryptosporidium meleagridis* | 18S | - | 8 | 28.57 | 7.91 | 2.67 | 6.91 | 5.88 | 10.32 | 4.44 |  | 12 | 57.14 | 7.49 | 3.56 | 6.47 | 4.81 | 10 | 5.19 |
| *Cryptosporidium microti* | 18S | - | 8 | 28.57 | 13.7 | 4.95 | 11.28 | 10.01 | 18.27 | 8.26 |  | 6 | 28.57 | 4.33 | 2.28 | 4.05 | 3.23 | 4.66 | 1.43 |
| *Cryptosporidium muris* | 18S | - | 8 | 28.57 | 10.29 | 3.69 | 9.88 | 7.03 | 12.36 | 5.33 |  | 11 | 52.38 | 7.44 | 3.33 | 5.71 | 5.3 | 9.12 | 3.82 |
| *Cryptosporidium parvum* | 18S | - | 28 | 100 | 40.16 | 18.63 | 35.63 | 31.92 | 40.94 | 9.02 |  | 21 | 100 | 32.88 | 8.56 | 31.31 | 26.87 | 35.32 | 8.45 |
| *Cryptosporidium ratti* | 18S | - | 3 | 10.71 | 7.15 | 3.6 | 6.43 | 5.2 | 8.74 | 3.55 |  | 7 | 33.33 | 3.91 | 2.49 | 3.42 | 2.17 | 5.23 | 3.05 |
| *Cryptosporidium suis* | 18S | - | - | - | - | - | - | - | - | - |  | 4 | 19.05 | 1.67 | 0.44 | 1.53 | 1.38 | 1.81 | 0.43 |
| *Cryptosporidium ubiquitum* | 18S | - | - | - | - | - | - | - | - | - |  | 4 | 19.05 | 2.42 | 0.38 | 2.55 | 2.31 | 2.66 | 0.35 |
| *Cryptosporidium wrairi* | 18S | - | 2 | 7.14 | 7.48 | 5.11 | 7.48 | 5.67 | 9.29 | 3.61 |  | 3 | 14.29 | 8.58 | 0.87 | 9.08 | 8.32 | 9.08 | 0.76 |
