# Supplementary material for: Multilocus Oxford Nanopore sequencing reveals genetic diversity of Giardia duodenalis and Cryptosporidium spp. in surface and wastewater in Southwestern Colombia
Source: Parasit Vectors. 2026 Aug 26;19:398. doi: 10.1186/s13071-026-07565-0 (PMC13587459; doi:10.1186/s13071-026-07565-0)
Supplement: Supplementary file 7 [file 13071_2026_7565_MOESM7_ESM.docx]

**Table S6.** Patterns of co-occurrence between *Cryptosporidium* species and *Giardia* assemblages detected in individual samples.

| **Sample** | **Number of taxa detected** | **Co-occurring taxa** |
| --- | --- | --- |
| P33 | 14 | *C. andersoni, C. canis, C. felis, C. hominis, C. meleagridis, C. microti, C. muris, C. parvum, C. ratti, C. ubiquitum, C. wrairi, G. duodenalis A, G. duodenalis B, G. duodenalis D* |
| P03 | 14 | *C. andersoni, C. canis, C. felis, C. hominis, C. meleagridis, C. microti, C. muris, C. parvum, C. ratti, C. ubiquitum, C. wrairi, G. duodenalis A, G. duodenalis B, G. duodenalis E* |
| P07 | 14 | *C. andersoni, C. canis, C. felis, C. hominis, C. meleagridis, C. microti, C. muris, C. parvum, C. ratti, C. suis, C. ubiquitum, G. duodenalis A, G. duodenalis B, G. duodenalis E* |
| P08 | 14 | *C. andersoni, C. canis, C. felis, C. hominis, C. meleagridis, C. microti, C. muris, C. parvum, C. ratti, C. suis, C. ubiquitum, G. duodenalis A, G. duodenalis B, G. duodenalis E* |
| P60 | 13 | *C. andersoni, C. canis, C. felis, C. hominis, C. meleagridis, C. microti, C. muris, C. parvum, C. ratti, C. suis, G. duodenalis A, G. duodenalis B, G. duodenalis E* |
| P02 | 11 | *C. andersoni, C. canis, C. felis, C. meleagridis, C. microti, C. muris, C. parvum, C. ratti, G. duodenalis A, G. duodenalis B, G. duodenalis E* |
| P80 | 11 | *C. andersoni, C. canis, C. felis, C. hominis, C. meleagridis, C. microti, C. muris, C. parvum, C. ratti, C. suis, C. ubiquitum* |
| P11 | 11 | *C. andersoni, C. canis, C. felis, C. meleagridis, C. microti, C. muris, C. parvum, C. ratti, G. duodenalis A, G. duodenalis B, G. duodenalis E* |
| P06 | 9 | *C. andersoni, C. canis, C. felis, C. meleagridis, C. muris, C. parvum, G. duodenalis A, G. duodenalis B, G. duodenalis E* |
| P55 | 8 | *C. andersoni, C. canis, C. parvum, C. wrairi, G. duodenalis A, G. duodenalis B, G. duodenalis D, G. duodenalis E* |
| P51 | 8 | *C. andersoni, C. canis, C. meleagridis, C. microti, C. muris, C. parvum, G. duodenalis B, G. duodenalis E* |
| P53 | 8 | *C. andersoni, C. canis, C. meleagridis, C. microti, C. muris, C. parvum, C. wrairi, G. duodenalis B* |
| P21 | 8 | *C. andersoni, C. canis, C. meleagridis, C. muris, C. parvum, C. wrairi, G. duodenalis A, G. duodenalis B* |
| P70 | 7 | *C. andersoni, C. canis, C. felis, C. meleagridis, C. microti, C. muris, C. parvum* |
| P98 | 7 | *C. andersoni, C. canis, C. felis, C. meleagridis, C. muris, C. parvum, C. ratti* |
| P05 | 7 | *C. andersoni, C. canis, C. meleagridis, C. muris, C. parvum, G. duodenalis A, G. duodenalis B* |
| P99 | 7 | *C. andersoni, C. canis, C. felis, C. meleagridis, C. muris, C. parvum, C. ratti* |
| P27 | 7 | *C. canis, C. parvum, G. duodenalis A, G. duodenalis B, G. duodenalis C, G. duodenalis D, G. duodenalis E* |
| P20 | 6 | *C. andersoni, C. canis, C. microti, C. parvum, G. duodenalis A, G. duodenalis B* |
| P12 | 6 | *C. andersoni, C. canis, C. microti, C. parvum, G. duodenalis A, G. duodenalis B* |
| P52 | 6 | *C. andersoni, C. canis, C. microti, C. parvum, G. duodenalis A, G. duodenalis B* |
| P41 | 6 | *C. andersoni, C. canis, C. parvum, G. duodenalis A, G. duodenalis B, G. duodenalis E* |
| P04 | 6 | *C. andersoni, C. canis, C. parvum, G. duodenalis A, G. duodenalis B, G. duodenalis E* |
| P31 | 6 | *C. andersoni, C. canis, C. parvum, G. duodenalis A, G. duodenalis B, G. duodenalis E* |
| P78 | 5 | *C. andersoni, C. canis, C. hominis, C. meleagridis, C. parvum* |
| P92 | 5 | *C. andersoni, C. canis, C. meleagridis, C. muris, C. parvum* |
| P82 | 5 | *C. andersoni, C. canis, C. meleagridis, C. muris, C. parvum* |
| P64 | 5 | *C. andersoni, C. canis, C. parvum, G. duodenalis A, G. duodenalis B* |
| P38 | 5 | *C. andersoni, C. canis, C. parvum, G. duodenalis A, G. duodenalis B* |
| P26 | 5 | *C. andersoni, C. canis, C. parvum, G. duodenalis A, G. duodenalis B* |
| P24 | 5 | *C. andersoni, C. canis, C. parvum, G. duodenalis A, G. duodenalis B* |
| P18 | 4 | *G. duodenalis A, G. duodenalis B, G. duodenalis D, G. duodenalis E* |
| P56 | 4 | *C. andersoni, C. canis, C. parvum, G. duodenalis B* |
| P65 | 4 | *C. andersoni, C. canis, C. muris, C. parvum* |
| P59 | 4 | *G. duodenalis A, G. duodenalis B, G. duodenalis D, G. duodenalis E* |
| P48 | 4 | *C. andersoni, C. parvum, G. duodenalis B, G. duodenalis E* |
| P39 | 4 | *C. andersoni, C. canis, C. meleagridis, C. parvum* |
| P15 | 3 | *G. duodenalis A, G. duodenalis B, G. duodenalis E* |
| P16 | 3 | *G. duodenalis A, G. duodenalis B, G. duodenalis E* |
| P54 | 3 | *G. duodenalis A, G. duodenalis B, G. duodenalis E* |
| P17 | 3 | *G. duodenalis A, G. duodenalis B, G. duodenalis E* |
| P28 | 3 | *G. duodenalis A, G. duodenalis B, G. duodenalis E* |
| P29 | 3 | *G. duodenalis A, G. duodenalis B, G. duodenalis E* |
| P45 | 3 | *G. duodenalis A, G. duodenalis B, G. duodenalis E* |
| P58 | 3 | *G. duodenalis A, G. duodenalis B, G. duodenalis E* |
| P14 | 3 | *G. duodenalis A, G. duodenalis B, G. duodenalis E* |
| P74 | 3 | *C. andersoni, C. canis, C. parvum* |
| P73 | 3 | *C. andersoni, C. canis, C. parvum* |
| P67 | 3 | *C. andersoni, C. canis, C. parvum* |
| P69 | 3 | *C. andersoni, C. canis, C. parvum* |
| P91 | 3 | *C. andersoni, C. canis, C. parvum* |
| P90 | 3 | *C. andersoni, C. canis, C. parvum* |
| P81 | 3 | *C. andersoni, C. canis, C. parvum* |
| P75 | 3 | *C. andersoni, C. canis, C. parvum* |
| P40 | 3 | *C. andersoni, C. canis, C. parvum* |
| P102 | 3 | *C. andersoni, C. parvum, G. duodenalis B* |
| P72 | 3 | *C. andersoni, C. canis, C. parvum* |
| P30 | 2 | *G. duodenalis A, G. duodenalis B* |
| P50 | 2 | *G. duodenalis A, G. duodenalis B* |
| P34 | 2 | *G. duodenalis A, G. duodenalis B* |
| P19 | 2 | *G. duodenalis A, G. duodenalis B* |
| P25 | 2 | *G. duodenalis A, G. duodenalis B* |
| P103 | 2 | *C. canis, C. parvum* |
| P13 | 2 | *G. duodenalis A, G. duodenalis B* |
| P01 | 2 | *G. duodenalis A, G. duodenalis B* |
| P57 | 2 | *G. duodenalis A, G. duodenalis B* |
| P62 | 1 | *C. parvum* |
| P66 | 1 | *C. parvum* |
| P95 | 1 | *G. duodenalis B* |
